# Mechanisms of host-bacterial interactions during *Escherichia coli* or *Staphylococcus aureus* infection of mammary epithelial cells

**DOI:** 10.3389/fvets.2025.1644768

**Published:** 2025-07-23

**Authors:** Zhengge Zhao, Zhiming Hou, Jianmin Chai, Chunfang Li, Tingyu Liu, Jianming Li, Shuyi Zhang, Lei Zhang, Yabin Ma

**Affiliations:** ^1^Guangdong Provincial Key Laboratory of Animal Molecular Design and Precise Breeding, School of Animal Science and Technology, Foshan University, Foshan, China; ^2^College of Animal Science and Veterinary Medicine, Shenyang Agricultural University, Shenyang, China; ^3^Hebei Animal Husbandry and Breeding Work Station, Shijiazhuang, China

**Keywords:** RNA-seq, bovine, Mac-T cells, *Escherichia coli*, *Staphylococcus aureus*, transcriptomics, PPI, mammary epithelial cells

## Abstract

**Introduction:**

Mastitis is one of the costliest diseases in the dairy industry. *Escherichia coli* (*E. coli*) and *Staphylococcus aureus* (*S. aureus*) are the two most predominant pathogens. However, the specific molecular mechanisms underlying the interactions between these pathogens and bovine mammary epithelial cells, especially for two pathogenic co-infections, remain poorly understood.

**Methods:**

Here, this study employed high-throughput RNA sequencing to comprehensively analyze the gene expression changes in bovine mammary epithelial cells upon individual and co-infection with *E. coli* and *S. aureus*.

**Results:**

Transcriptomic analysis identified 282 differentially expressed genes (DEGs) in the *E. coli*-infected group (E group), with 246 upregulated and 36 downregulated genes. Notably, pro-inflammatory genes (*CXCL8*, *GRO1*, *CCL20*) were significantly induced, and functional enrichment analysis demonstrated robust activation of inflammatory pathways including *TLR*/*NF-κB* and *IL-17* signaling cascades. In contrast, the *S. aureus*-infected group (S group) exhibited 354 DEGs (314 upregulated, 40 downregulated), featuring pathogen-specific upregulated genes (*ESM1*, *IL18RAP*). Functional annotation revealed predominant involvement of metabolic processes, particularly ATP metabolism and chaperone complex activities. The co-infection group (ES group) displayed 307 DEGs (277 upregulated, 30 downregulated), demonstrating a unique “inflammatory-metabolic” dual-mode signature that integrated inflammatory features from the E group with metabolic reprogramming characteristics of the S group. Protein-protein interaction network analysis further delineated pathogen-specific hubs: inflammatory mediators (CXCL8, CCL20, IL6) in the E group, molecular chaperones (CCT5, RUVBL1/2) in the S group, and a distinctive IL6-FBL-centered network in co-infection. These findings elucidate pathogen-specific molecular mechanisms at the transcriptomic level, particularly revealing a unique “inflammatory-metabolic” dual-mode regulatory network during co-infection states. These findings provide new insights into the pathogenesis of mastitis and provide a theoretical basis for developing targeted prevention and control strategies.

## 1 Introduction

Bovine mastitis, a common disease threatening the healthy development of the global dairy industry, causes enormous economic losses annually. It is estimated that the direct economic losses caused by clinical mastitis alone reach hundreds of dollars per cow (1). In the meantime, the impact of subclinical mastitis is even more widespread, such as reducing milk yield and quality (2, 3), and increasing antibiotic usage (4), thereby raising public health concerns about food safety and bacterial resistance (5). The etiology of mastitis involves factors such as feeding management, environmental conditions, and pathogenic microorganisms, with bacterial infections being the primary cause. Among the various pathogens, *Escherichia coli* (*E. coli*) and *Staphylococcus aureus* (*S. aureus*) are representative. Current research has found that their infection characteristics differ: *E. coli* primarily causes acute clinical mastitis, whereas *S. aureus* tends to lead to chronic and persistent infections. The two pathogens exhibit significant differences in pathogenic mechanisms and host responses (6). These differences suggest that during the invasion of bovine mammary epithelial cells, the two pathogens may activate distinct host recognition receptors (such as *TLR4* and *TLR*2), regulate different cellular signaling transduction networks (such as the *NF-κB* and *MAPK* pathways), and ultimately induce specific immune response patterns in the host cells (7).

In recent years, with the advancement of molecular and cellular biology technologies, significant progress has been made in research on the interactions between mammary epithelial cells and pathogenic bacteria. Currently, studies on the molecular mechanisms of single infections by *E. coli* or *S. aureus* have been relatively well-established. *E. coli* primarily activates the Toll-like receptor 4 (*TLR4*) signaling pathway via lipopolysaccharide (LPS), triggering a strong inflammatory response (8). In contrast, *S. aureus* disrupts epithelial barrier function through virulence factors, such as α-hemolysin and demonstrates stronger intracellular parasitism (9). Multiple studies have also found that nutrient-sensing regulators play an important role in alleviating bacteria-induced inflammatory responses. Studies have shown that selenomethionine (10–12) and cis-9, trans-11 conjugated linoleic acid (13) mitigate *E. coli*-induced inflammation through distinct mechanisms. Furthermore, *PINK1*/Parkin-mediated mitophagy (14) and selenium-mediated ROS modulation (15) have been found to attenuate *NLRP3* inflammasome activation (16) triggered by *S. aureus* in bovine mammary epithelial cells. While extensive research has focused on infections caused by either pathogen individually, the synergistic interaction mechanisms of these two bacterial co-infections that are usually observed in industry remain poorly understood.

Although numerous studies have investigated single-pathogen infection mechanisms, the regulatory effects of *E. coli* and *S. aureus* on host cells at the transcriptomic level—particularly their synergistic or antagonistic interactions during co-infection—remain poorly understood. Several critical questions are needed to be addressed: (1) How do these two pathogens differentially modulate host gene expressions to evade immune defenses? (2) Whether unique transcriptional regulatory patterns emerge during co-infection? (3) How does the infection dose influence these interactions? To address these gaps, this study employs transcriptomic sequencing to delineate the molecular interaction networks in bovine mammary epithelial cells during mono- or co-infection with *E. coli* and *S. aureus*. By elucidating both pathogen-host and pathogen-pathogen interaction mechanisms, our findings will provide theoretical foundations and experimental evidence for understanding mastitis pathogenesis and developing more effective prevention strategies.

## 2 Materials and methods

### 2.1 Experimental design

A systematic experiment was designed to investigate the host-pathogen interaction mechanisms. The bovine mammary epithelial cells (Mac-T cell line) were challenged by blank, *Escherichia coli*, *Staphylococcus aureus* and both pathogens. At the same time, Mac-T cells were subjected to mono- or co-infection at three different multiplicity of infection ratios (10:1, 1:1, and 1:10) to simulate varying infection intensities (group-named as 1, 2, and 3, respectively). Each infection ration had 4 replicates.

### 2.2 Cell culture and pathogen stimulation

This study utilized Mac-T cells (a bovine mammary epithelial cell line) obtained from the Shanghai Cell Bank. After thawing from liquid nitrogen storage, the cells were cultured in complete medium consisting of 89% DMEM basal medium, 10% fetal bovine serum (FBS), and 1% penicillin/streptomycin (double antibiotics). The cells were maintained in a constant temperature incubator at 37°C with 5% CO₂. To ensure experimental stability and reproducibility, the MAC-T cells were passaged for 3 consecutive generations (approximately 48 h per passage) prior to formal experiments to establish a homogeneous and well-conditioned cell population. During this period, cell morphology and growth status were regularly observed under an inverted microscope to avoid contamination or abnormal growth. Once cell growth stabilized, the cells were digested with 0.25% Trypsin–EDTA to prepare a uniform cell suspension for subsequent experiments.

For the preparation of *Escherichia coli* and *Staphylococcus aureus*, 0.5–0.8 mL of LB liquid medium was pipetted into the lyophilized bacterial ampoules, mixed thoroughly, and inoculated into liquid medium. The cultures were then incubated at 37°C with shaking at 150 rpm for 24 h. A 100 μL aliquot of the bacterial suspension was streaked onto TSA plates and incubated at 37°C for 24 h. Single colonies were picked up and inoculated into LB liquid medium, followed by 12 h of shaking at 37°C for subsequent use. The bacterial suspension was then transferred to 100 mL of LB liquid medium, and samples were collected at 0, 1.5, 3, 4, 6, 8, 10, 14, 16, and 20 h to measure the OD₆₀₀ and perform plate counting, establishing the bacterial growth curve.

MAC-T cells were seeded into 6-well plates, and when cell density reached 80%, they were washed three times with sterile phosphate-buffered saline (PBS) and switched to serum-free, antibiotic-free DMEM basal medium for a 2-h incubation at 37°C. Bacterial suspensions of *E. coli*, *S. aureus*, or a mixture of both pathogens were added at multiplicity of infection (MOI) ratios of 10:1, 1:1, and 1:10, respectively, followed by incubation at 37°C with 5% CO₂ for 2 h. After discarding the culture medium, the cells were washed three times with pre-warmed PBS. The cells were then incubated for 1 h in complete medium containing gentamicin (50 μg/mL) to eliminate extracellular bacteria. Following incubation, the cells were washed three additional times with pre-warmed PBS to completely remove residual gentamicin. Finally, the cells were cultured in fresh complete medium supplemented with 5 μL of double antibiotics (penicillin/streptomycin) for further maintenance. The culture medium from the inflammatory and control groups was collected in sterile EP tubes for further analysis. Additionally, a portion of the inflammatory and control cells were lysed to obtain cell lysates. The collected culture medium and cell lysates were spread onto LB plates and incubated at 37°C for 24 h to assess the successful establishment of the inflammation model. If the model was successfully established, no bacterial colonies would grow in the culture medium, while colonies would be observed in the inflammatory cell lysates, with no colonies present in the control group lysates.

### 2.3 RNA extraction, and transcriptomics sequencing

This study extracted total RNA from MAC-T cells using TRIzol reagent (Takara, Beijing, China) following the manufacturer’s standard protocol. All procedures were performed on ice with strict time control to minimize RNA degradation. The extracted RNA samples were immediately dissolved in RNase-free water, and their concentration, purity, and integrity (RIN > 7.0) were assessed using a Nanodrop spectrophotometer and agarose gel electrophoresis. Qualified samples were sent to Novogene Co., Ltd. (Tianjin China) for RNA-seq library preparation and sequencing analysis. RNA sequencing was performed on the Novaseq-PE150 platform to generate 150 aired end reads.

### 2.4 Bioinformatics

Raw sequencing data were processed using Trimmomatic (v0.39) (17) with the following parameters: Phred+33 quality encoding format; sliding window trimming (SLIDINGWINDOW:4:20) where reads were trimmed when the average base quality in a 4-bp window fell below 20; minimum length threshold (MINLEN:60) where reads shorter than 60 bp after trimming were discarded. The resulting high-quality reads (clean data) were used for subsequent analysis. The bovine reference genome (Bos_taurus. ARS-UCD1.3) FASTA file (Bos_taurus. ARS-UCD1.3.dna_sm.toplevel.fa) was downloaded from Ensembl database, and genome indexes were built using HISAT2’s hisat-build tool for sequence alignment. The corresponding GFF3 annotation file (Bos_taurus. ARS-UCD1.3.113.gff3) was also downloaded for transcript assembly and gene annotation. Quality-controlled clean data were aligned to the reference genome using HISAT2 (18) (v2.2.1). Paired-end sequencing data from each sample were aligned to generate SAM format files, which were then converted to BAM format using SAMtools (19). The BAM files were subsequently sorted for downstream analysis. Transcript assembly was performed on the sorted BAM files using StringTie (20) (v2.2.1). Initial transcript assembly was conducted using both BAM files and GFF3 annotation files, generating GTF format files containing detailed transcript information and gene expression levels. A sample_list.txt file was created containing sample names and corresponding GTF file paths. Finally, a Python script was used to process the StringTie-generated GTF files and produce a gene count matrix (gene_count_matrix.csv).

### 2.5 Statistics

Principal component analysis (PCA) was performed using ImageGP (21).^1^ The alpha level for determining the significance of PCA separation in our study was set at 0.05. To assess the significance of the separation, we employed the PERMANOVA (Permutational Multivariate Analysis of Variance) test. In RStudio (2023.6.0.421), we conducted differential expression analysis by first mapping gene IDs from gene_count_matrix.csv to the ARS-UCD1.3 database using BioMart on Ensembl^2^ (22). Using DESeq2 (23) (v1.46.0), we compared experimental groups (E1, E2, E3, ES1, ES2, ES3, S1, S2, S3) against control group DZ (as denominator), with differential genes identified by Padj<0.05 & |log2FoldChange| ≥ 1. Volcano plots were generated using ggplot2 (v3.5.2). Differentially expressed genes were analyzed in STRING (24)^3^ with *Bos taurus* selected as organism and minimum interaction score set to 0.7. We performed GO and KEGG enrichment analyses and generated protein–protein interaction (PPI) networks. PPI networks were visualized in Cytoscape (25) (v3.10.3) using Analyze Network tool. Nodes were colored and sized by Degree score (darker/larger indicating higher degree), with cluster annotations showing functional descriptions. All figures were finalized and assembled using Adobe Illustrator (v2025).

## 3 Results

### 3.1 Transcriptomics characteristics

The transcriptome sequencing of 39 Bovine Mammary Epithelial Cell (BMEC) samples generated a total of 791,931,172 raw reads. After quality control, 756,830,895 high-quality reads were successfully aligned to the *Bos taurus* reference genome (ARS-UCD1.3, NCBI) with an overall alignment rate exceeding 94% for all samples (Supplementary Table S1). All samples were subjected to principal component analysis (PCA) (Supplementary Figure S1). There were highly significant differences between the control group (Control) and the three concentration groups of *Escherichia coli* infection (E1, E2, E3) (*p* < 0.01). The Control group showed significant differences with the high-concentration *Staphylococcus aureus* infection group (S1) and the medium-concentration *Staphylococcus aureus* infection group (S2) (*p* < 0.05). Additionally, the Control group exhibited a significant difference with the low-concentration co-infection group of the two pathogens (ES3) (*p* < 0.05) (Supplementary Table S2).

### 3.2 Different pathogens stimulated the changes of expressed genes in Mac-T cells

To characterize the different expressed genes (DEGs) among treatment, DESeq2 was performed. Firstly, we compared pathogenic challenged treatments and the control group (Control). Compared to Control, the *Escherichia coli*-infected group (E) had identified 282 DEGs including 246 upregulated and 36 downregulated genes (Supplementary Table S3). The *Staphylococcus aureus*-infected group (S) showed 354 DEGs (314 upregulated and 40 downregulated). For the co-infection group (ES), 307 DEGs were detected (277 upregulated and 30 downregulated). Next, the DEGs from different infection ratios of each treatment were also classified. Using Control as baseline, the high- (E1) and medium-concentration (E2) infection subgroups within *Escherichia coli*-infected group (E) exhibited the highest number of DEGs (291 and 303, respectively). In S, the medium-concentration subgroup (S2) had the greatest numbers of DEGs, suggesting this concentration caused the biggest alteration of Mac-T cells. Lastly, for the co-infection group (ES), the high-concentration subgroup (ES1) had the highest amount of DEGs (366), followed by medium- (ES2) and low-concentration (ES3) subgroups.

**Figure 1:**
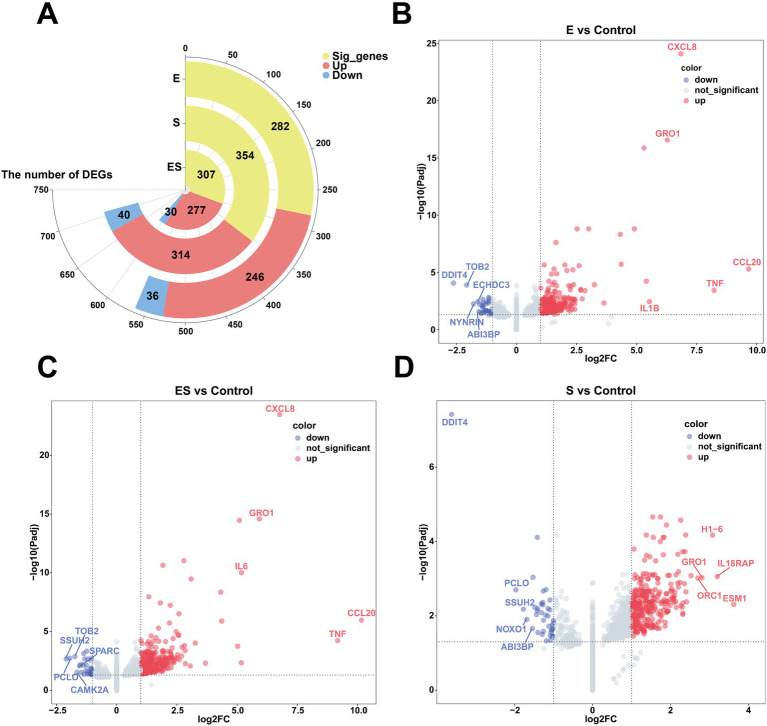
Differentially expressed genes in pathogen-stimulated Mac-T Cells. **(A)** Shows a circular bar plot of significant genes with segments for pregulated (red) and downregulated (blue) genes in Escherichia coli (E), Staphylococcus aureus (S), and co-infection (ES) groups. **(B–D)** Depict volcano plots comparing gene expression changes between experimental groups vs. control. Red dots indicate upregulated genes, blue dots indicate downregulated genes, and gray dots are not significant. Key genes like CXCL8 and TNF are highlighted. **(B)** Compares E vs. Control, **(C)** Compares ES vs. Control, and **(D)** Compares S vs. Control.

The Volcano plots were generated to visualize DEGs across treatment groups, with the top 5 upregulated and downregulated genes labeled (Figure 1; Supplementary Figure S2; Supplementary Table S4). Our results showed consistent upregulated DEGs in E and ES treatment groups regardless of the concentration of pathogens. For example, *CXCL8* (*IL-8*, neutrophil chemoattractant), *GRO1* (growth-regulated oncogene involved in inflammation), *CCL20* (immune cell recruiter), *TNF* (core inflammatory mediator), and *IL1B*/*IL6* (key pro-inflammatory cytokines) were upregulated genes in E, E1-E3, ES, ES1-ES3 compared to Control, suggesting a robust immune response to *E. coli* infection. However, concentration-dependent effects were observed: in *E. coli* infection, *IL6* was upregulated in E1 while *CSF2* (granulocyte-macrophage colony-stimulating factor) was upregulated in E3. Interestingly, top upregulated DEGs in S treatment were significantly different. In addition, similar patterns of downregulated genes were also observed. *DDIT4* (DNA damage/stress regulator) consistently appeared across multiple groups (E, E1-E3, ES, S, S1-S3), potentially indicating bacterial infection-induced cellular stress or metabolic suppression. For *S. aureus* infection, *WSB1* (ubiquitination-related) was downregulated in S1, while only *DDIT4* was downregulated in S3, suggesting broader impacts on stress pathways at higher infection concentrations.

### 3.3 Functional enrichment analysis of differentially expressed genes in pathogen-stimulated Mac-T cells

**Figure 2:**
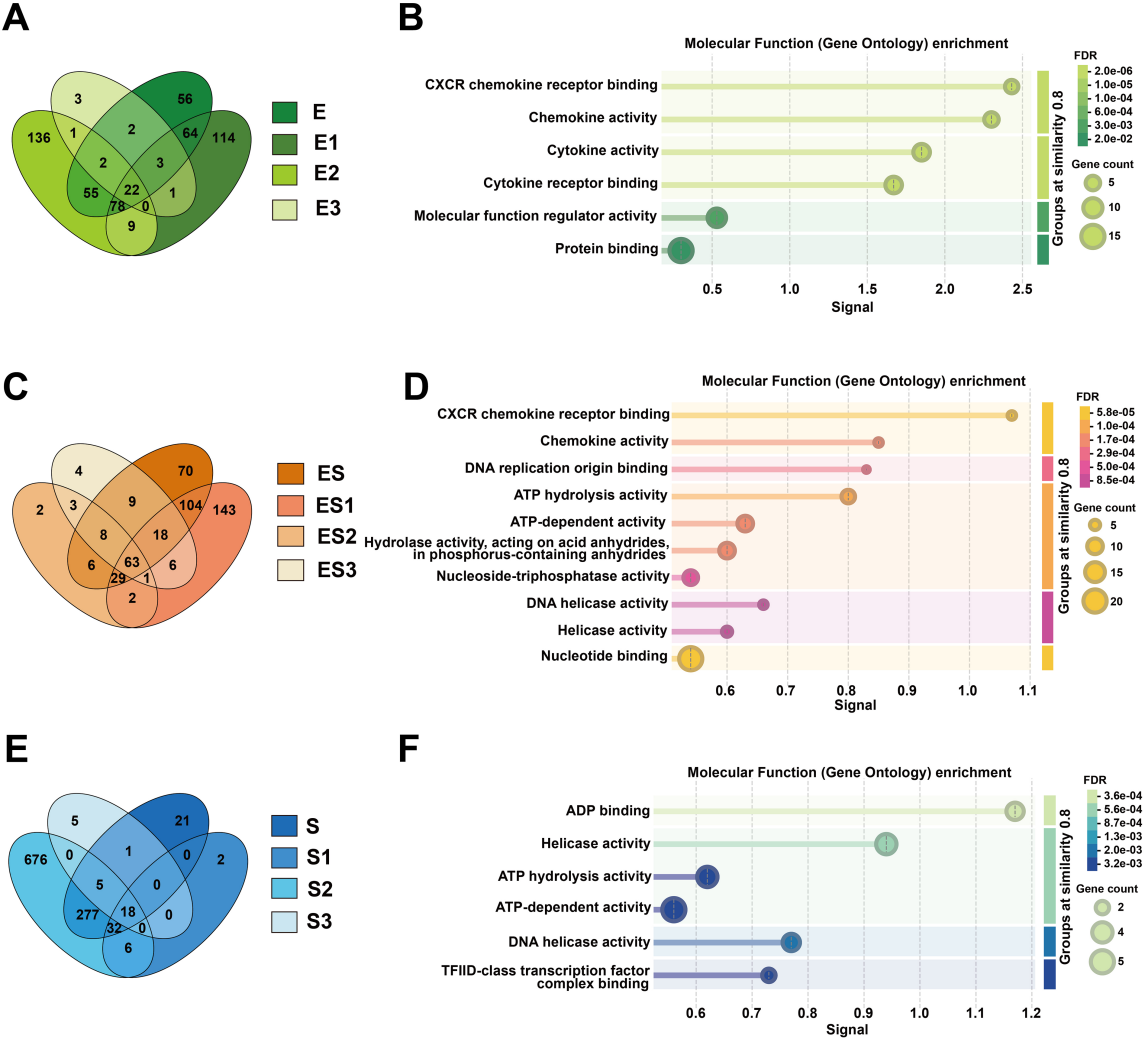
Comparative analysis of shared differentially expressed genes (DEGs) and their molecular functions across infection groups. **(A)** Shows a green Venn diagram comparing DEG overlap among Escherichia coli subgroups (E, E1, E2, E3), while **(C)** displays an orange Venn diagram for co-infection subgroups (ES, ES1, ES2, ES3), and **(E)** presents a blue Venn diagram for Staphylococcus aureus subgroups (S, S1, S2, S3). The corresponding molecular function enrichment results from Gene Ontology analysis are shown in **(B, D, F)**: **(B)** illustrates enriched functions like chemokine activity in E subgroups, **(D)** highlights DNA helicase activity in ES subgroups, and **(F)** identifies ADP binding and ATP hydrolysis activities in S subgroups.

Venn diagram analysis of differentially expressed genes (DEGs) revealed shared DEGs across different rations, such as 22 genes in *E. coli* infection, 18 genes in *S. aureus* infection, and 63 genes in co-infection treatment (Figure 2). Functional enrichment analysis of these shared DEGs is presented in Figure 2. For molecular function (MF) enrichment, significant enrichments of *CXCR* chemokine receptor binding and chemokine activity were found in all subgroups of both the *E. coli* and the co-infection treatments. Similarly, the co-infection group and subgroups in *S. aureus* were both enriched for ATP energy metabolism-related functions and nucleic acid (DNA/RNA) metabolic activities, such as ATP hydrolysis activity and DNA helicase activity. In biological process (BP) enrichment analysis, both the *E. coli* and co-infection groups were enriched for multiple immune defense pathways, including cellular response to lipopolysaccharide, chemokine-mediated signaling pathway, and cellular response to cytokine stimulus. Cellular component (CC) analysis demonstrated that the *S. aureus* and co-infection groups were enriched for nuclear structures such as nucleoplasm and nuclear chromosome, as well as chaperone complex. KEGG pathway enrichment analysis of the *E. coli* and co-infection groups identified shared immune-related signaling pathways, including the *IL-17* signaling pathway, *NF-kappa B* signaling pathway, and Toll-like receptor signaling pathway (Supplementary Figures S6, S8, S10).

Furthermore, we identified unique genes specific to different concentrations of *E. coli* infection. The E3 subgroup contained three distinctive genes: *HBEGF* (heparin-binding epidermal growth factor), *PDCD7* (programmed cell death protein 7), and *SMTNL2* (smooth muscle tropomyosin light chain phosphatase regulatory subunit 2). The E1 and E2 subgroups exhibited 114 and 136 unique genes, respectively (Supplementary Figure S3). Functional enrichment analysis of E1 and E2 revealed that both subgroups were associated with rRNA metabolic processes, rRNA processing, and ribonucleoprotein complex biogenesis in terms of biological processes (BP). However, E1 was more enriched in nucleic acid metabolic processes, RNA processing, and RNA metabolic processes, while E2 showed stronger associations with telomere maintenance and chromosome organization. For cellular components (CC), both subgroups were localized to the nucleolus and intracellular organelle lumen, consistent with rRNA processing and ribosome synthesis sites. Additionally, E1 was enriched in spliceosome-related complexes (e.g., U4/U6 x U5 tri-snRNP complex), methylation-related complexes (e.g., methyltransferase complex), and proteasome regulatory particles. In contrast, E2 was enriched in preribosomes, mitochondria, fibrillar centers, and chaperonin-containing T-complexes. Regarding molecular functions (MF), E1 was associated with proteasome-activating activity, whereas E2 was primarily linked to translation factor activity (e.g., translation initiation factor activity, RNA binding) and energy metabolism (e.g., ATP hydrolysis activity, electron transfer activity). KEGG pathway analysis indicated that both subgroups participated in neurodegenerative diseases and oxidative phosphorylation. E1 specifically showed enrichment in RNA degradation and spliceosome pathways, while E2 was uniquely associated with RNA transport and thermogenesis (Supplementary Figures S4, S5).

We also observed that different concentrations of *S. aureus* infection exhibited unique sets of DEGs. Specifically, the S1 subgroup contained two distinctive genes (*AIRIM* and *ZBTB26*), while the S3 subgroup had five unique genes (*DUSP5*, *GEMIN4*, *HBEGF*, *TAF5*, and *TRMT1*). Notably, the S2 subgroup showed the highest number of unique DEGs (676 genes) (Supplementary Figure S3). Functional enrichment analysis of S2-specific DEGs revealed predominant associations with mitochondrial respiratory chain and energy metabolism pathways. These included mitochondrial respiratory chain complex assembly, mitochondrion organization, mitochondrial respirasome, and oxidative phosphorylation (Supplementary Figure S9).

Regarding the co-infection of cells by *E. coli* and *S. aureus*, the unique genes of ES2 are *DZIP1* and *SMTNL2*; the unique genes of ES3 are *ALDH4A1*, *DNASE1L1*, *FAM227A*, and *KAT2B*; and ES1 has 143 unique genes (Supplementary Figure S3). Functional enrichment analysis of the unique differentially expressed genes in ES1 revealed that in terms of BP, they are primarily enriched in nucleic acid metabolism and protein metabolic regulation. In terms of CC, they are mainly enriched in chaperonin complexes and nuclear structures. In terms of MF, they are primarily enriched in protein folding and nucleic acid binding. In terms of KEGG, all pathways are related to neurodegenerative diseases and involve protein homeostasis imbalance (Supplementary Figure S7).

### 3.4 PPI network analysis of differentially expressed genes in pathogen-stimulated Mac-T cells

**Figure 3:**
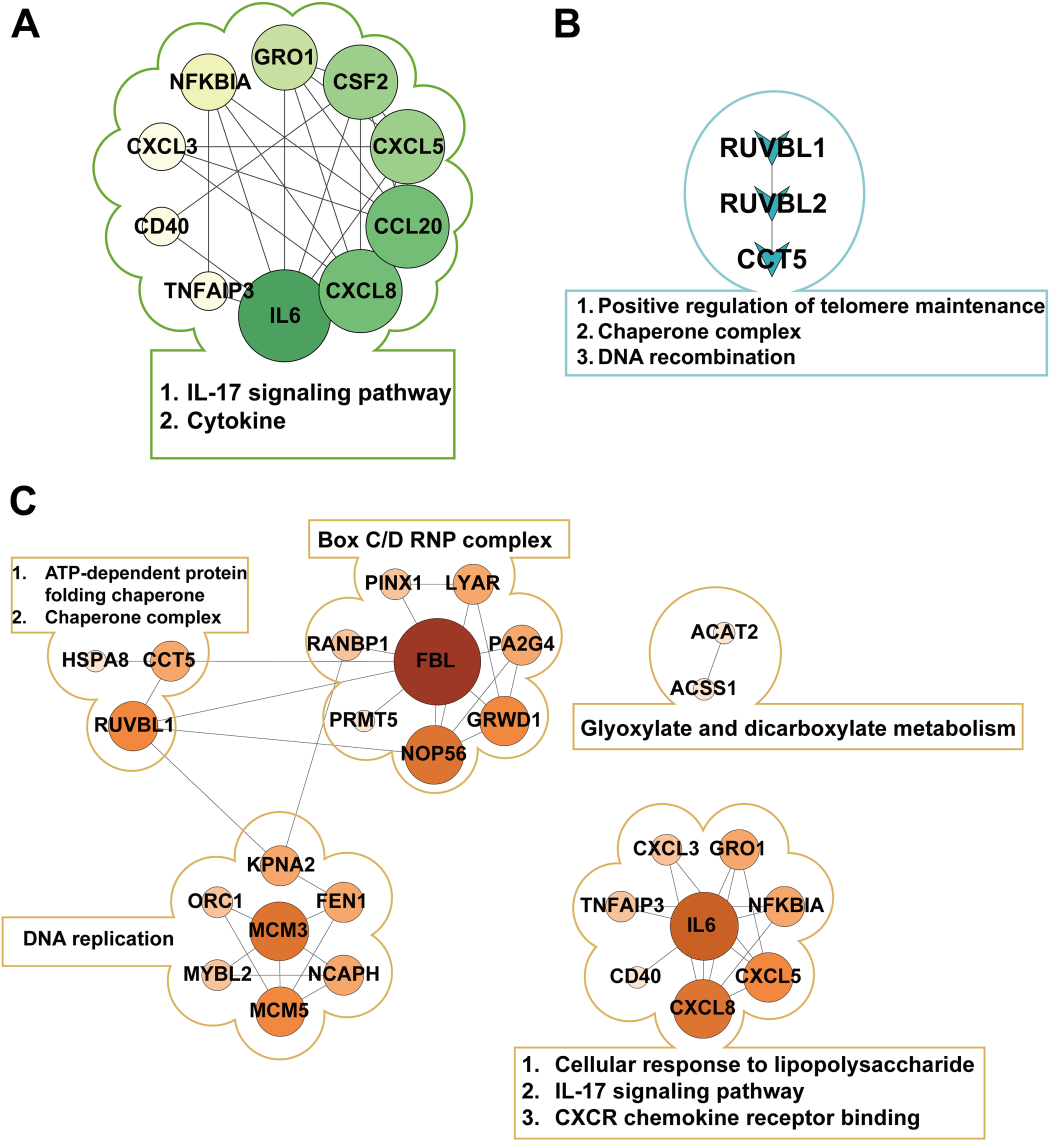
Protein-protein interaction (PPI) networks in different pathogenic bacterial infection groups. **(A)** Shows the PPI network of E1-, E2-, E3-, and E-shared genes in the Escherichia coli infection group, with IL6 as the central hub. **(B)** Displays the PPI network of S1-, S2-, S3-, and S-shared genes in the Staphylococcus aureus infection group, highlighting the key proteins RUVBL1, RUVBL2, and CCT5. **(C)** Presents the PPI network of ES1-, ES2-, ES3-, and ES-shared genes in the co-infection group, forming three distinct clusters with FBL and IL6 as core nodes.

Protein–protein interaction (PPI) network analysis was performed using STRING database with a minimum interaction score threshold of 0.7, revealing distinct network topologies across treatment groups (Figure 3; Supplementary Table S5). In the E group, the PPI network exhibited CXCL8, CCL20, and IL6 as central hub nodes, highlighting their pivotal roles in inflammatory responses. Conversely, the S group displayed CCT5, RUVBL1, and RUVBL2 as primary hub nodes, indicative of their involvement in protein folding and chromatin remodeling processes. Notably, k-means clustering of the ES group PPI network identified three functional modules, with IL6 and FBL emerging as the most prominent hub nodes, suggesting their critical involvement in the host response to polymicrobial infection.

## 4 Discussion

In this study, we first performed differential gene expression analysis on bovine mammary epithelial cells infected with *Escherichia coli* (*E. coli*) and *Staphylococcus aureus* (*S. aureus*). According to the DESeq2 results, the number of differentially expressed genes (DEGs) varied across different treatment groups. In the single-infection groups, *S. aureus* infection (S group) induced more DEGs than *E. coli* infection (E group) (354 vs. 282), which may reflect *S. aureus*’s more complex virulence factor repertoire and stronger immune activation capacity. Meanwhile, the co-infection group (ES group) had 307 DEGs, an intermediate number between the two single-infection groups, suggesting that the effect was not simply additive and indicating potential interactions or competition between the two pathogens during co-infection. This aligns with the findings of Li et al. (26), who demonstrated through QTL combinations that *E. coli* and *S. aureus* indeed interact during co-infection. Across all groups, the number of upregulated genes exceeded that of downregulated genes, indicating that the host’s primary response to bacterial infection involves gene activation rather than suppression (27, 28). Additionally, based on concentration analysis, we found that the number of DEGs in the high-concentration (E1) and medium-concentration (E2) *E. coli* infection groups was nearly 10 times higher than in the low-concentration (E3) group, suggesting a stronger response of bovine mammary epithelial cells to E1 and E2. Similarly, the medium-concentration *S. aureus* infection group (S2) exhibited over 1,000 DEGs (1,014), which were 17 times higher than the high-concentration group (S1) and 35 times higher than the low-concentration group (S3), indicating an extremely strong response of bovine mammary epithelial cells to S2. Furthermore, the response to the high-concentration co-infection group (ES1) was stronger than that of the medium- (ES2) and low-concentration (ES3) groups.

Furthermore, we analyzed the TOP5 upregulated DEGs in bovine mammary epithelial cells infected with pathogenic bacteria. *CXCL8* (*IL-8*), a potent neutrophil chemoattractant, strongly activates neutrophil migration to infection sites through its receptors *CXCR1* and *CXCR2* (29), while *GRO1* (*CXCL1*) has been established as a key mediator in bovine mastitis pathogenesis (30, 31). The classic proinflammatory cytokines *TNF* and *IL-1B* amplify inflammatory signals via the *NF-κB* pathway (32), and *CCL20* enhances mucosal immunity by recruiting Th17 cells (33). Our study found these factors significantly upregulated in *E. coli*-infected bovine mammary epithelial cells, likely due to robust LPS-*TLR4*-*NF-κB* pathway activation, where bacterial lipopolysaccharide (LPS) engages *TLR4* to drive expression of downstream inflammatory mediators (*TNF*, *IL-1B*) and chemokines (*CXCL8*, *GRO1*, *CCL20*) (34, 35). In contrast, *S. aureus* infection preferentially upregulated distinct molecular signatures: *ESM1* [an endothelial marker regulating vascular permeability via VEGF (36)], *IL18RAP* [indicating *NLRP3* inflammasome activation (37, 38)], the histone variant *H1-6* [involved in chromatin remodeling (39)], and DNA replication initiator *ORC1*, demonstrating reliance on the *IL18RAP*-*NLRP3*-*IL18* axis for cell-mediated immunity alongside effects on epigenomic regulation, cell cycle progression, and vascular dynamics. Notably, during co-infection, *IL6* replaced *IL-1B* among the top five upregulated genes, suggesting a shift toward *IL6*-*STAT3* mediated inflammation (40). *CCL20* recruits Th17 cells and dendritic cells through the *CCR6* receptor, activating mucosal immune defense (41). *IL6* drives the differentiation of Th17 cells, forming a positive feedback loop with *CCL20* to enhance antimicrobial peptide secretion (42). This establishes a “mucosal-systemic immune bridge” to cope with the special microenvironment of mixed infections. The persistent high expression of *GRO1* indicates that the neutrophil response is doubly enhanced (43), which may exacerbate cell damage.

Additionally, we analyzed the TOP5 downregulated DEGs in bovine mammary epithelial cells in response to pathogenic bacterial infection. *DDIT4* regulates cellular autophagy and metabolism by inhibiting mTORC1 (44), while *TOB2* modulates mRNA degradation (45). In our study, predominant downregulation of *ECHDC3*, *DDIT4*, and *TOB2* in the *E. coil* group suggests Gram-negative bacteria reprogram host metabolism by suppressing fatty acid oxidation (46) and activating *mTOR* pathways, while simultaneously prolonging inflammatory cytokine mRNA half-life to amplify immune responses. The observed downregulation of *NOXO1* [a NADPH oxidase component regulating ROS production (47)] and *PCLO* (a synaptic activity regulator involved in neural signaling) in the S group indicates potential bacterial strategies to minimize tissue damage by reducing ROS generation and modulating neuro-immune crosstalk to control local inflammation. Notably, the conserved downregulation of *ABI3BP* [an extracellular matrix regulator inhibiting cell migration (48–50)] and *DDIT4* across both E and S groups reveals pathogen-independent adaptive strategies involving Extracellular Matrix degradation and metabolic reprogramming. The ES group exhibited distinctive downregulation of *SPARC* [potentially accelerating tissue repair while increasing bacterial dissemination risk through impaired matrix remodeling (51)] and *CAMK2A* [disrupting epithelial barrier function via calcium signaling interference (52)], collectively establishing a unique “high-inflammation/low-repair” microenvironment. The persistent downregulation of *TOB2* and *PCLO* in ES group further reinforces synergistic effects between sustained inflammatory signaling and suppressed neural regulation, presenting a non-additive gene regulatory pattern that identifies precise molecular targets for intervention in polymicrobial infections. These findings reveal conserved and pathogen-specific mechanisms of host cell reprogramming during mastitis progression.

Through comparative GO and KEGG enrichment analyses of the *E. coli* infection group, *S. aureus* infection group, and *E. coli* and *S. aureus* group, significant differences in molecular features among the three infection modes were observed. For the *E. coli* infection group, at the biological process (BP) level, the most prominent feature was the robust activation of the “cellular response to lipopolysaccharide,” directly reflecting the core mechanism by which *E. coli* triggers host immune responses via its characteristic LPS component. This process further initiated cascading reactions, including “neutrophil chemotaxis” and “chemokine-mediated signaling pathway,” aligning with the well-documented pathological hallmark of *E. coli* infection—massive neutrophil infiltration (53–55). Notably, the activation of the “antimicrobial humoral immune response mediated by antimicrobial peptides” suggested the simultaneous engagement of nonspecific defense mechanisms, forming a multi-layered immune protection network. In terms of molecular function (MF), the enrichment of “*CXCR* chemokine receptor binding” and “cytokine activity” stood out, providing a molecular interaction-based explanation for the execution of these biological processes. Importantly, these molecular functions perfectly corresponded to the neutrophil chemotaxis and inflammatory responses in BP, constituting a complete signaling cascade from recognition to effector response. KEGG pathway analysis further enriched our understanding. The enrichment of the “*NF-κB* signaling pathway” and “Toll-like receptor signaling pathway” confirmed the central role of the *TLR4*-*NF-κB* axis in *E. coli* recognition (56), while the activation of the “*IL-17* signaling pathway” revealed the critical involvement of Th17 cell-mediated mucosal immune defense in this process (57). Interestingly, the enrichment of disease-related pathways such as “rheumatoid arthritis” and “transcriptional dysregulation in cancer” may imply pathological consequences resulting from prolonged or recurrent infections. These analytical results mutually corroborate, collectively delineating a comprehensive molecular map of *E. coli* infection: LPS activates the *NF-κB* pathway via *TLR4*, which leads to the massive secretion of pro-inflammatory cytokines and chemokines. This in turn results in neutrophil recruitment and activation, followed by antimicrobial peptide release and Th17 cell-mediated mucosal immune defense. While this process effectively combats infection, it may also lead to tissue damage and chronic inflammation risks.

For the *S. aureus* infection group, at the molecular function (MF) level, the significant enrichment of energy metabolism-related functions such as “ATP hydrolysis activity” and “DNA helicase activity” indicated that various toxins secreted by *S. aureus* disrupted host cell energy metabolism, while host cells initiated DNA damage repair mechanisms in response to infection pressure (58). Additionally, the enrichment of “TFIID-class transcription factor complex binding” suggested that *S. aureus* may regulate host gene expression by interfering with basal transcriptional machinery, a strategy contrasting with *E. coli*’s direct activation of inflammatory pathways via *TLR4*. In the cellular component (CC) category, the marked enrichment of protein folding-related structures such as “chaperone complex” and “R2TP complex” implied that host cells were coping with protein misfolding stress induced by bacterial toxins, while *S. aureus* might exploit host chaperone systems to facilitate proper folding of its own proteins. Concurrently, the enrichment of multiple chromatin-modifying complexes, including the “NuA4 histone acetyltransferase complex” and “MLL1 complex,” indicated that *S. aureus* infection might induce broad epigenetic modifications. These findings collectively outline the molecular signature of *S. aureus* infection: bacterial toxins disrupt host energy metabolism and protein homeostasis, which in turn activates chaperone systems and DNA repair mechanisms. This also interferes with transcriptional machinery and epigenetic regulation, ultimately leading to host cell dysfunction. Unlike the intense inflammatory response triggered by *E. coli*, *S. aureus* tends to achieve infection through “metabolic interference” and “epigenetic modulation.”

In the case of co-infection by *E. coli* and *S. aureus*, host cells exhibit complex molecular response mechanisms. These mechanisms involve the recognition and response to pathogens, including the activation of cells’ responses to lipopolysaccharides and chemokines through pattern recognition receptors such as *TLR4*, as well as the subsequent inflammatory responses and immune regulation triggered. Host cells facilitate the chemotaxis and recruitment of neutrophils by activating *CXCR* chemokine receptor binding and chemokine activity, while also modulating inflammatory responses through *NF-κB*, *TNF*, and *IL-17* signaling pathways. Additionally, host cells undergo metabolic reprogramming, reflected in the regulation of nucleic acid and macromolecule metabolic processes, as well as the activation of energy metabolism-related pathways such as glyoxylate and dicarboxylate metabolism and pyruvate metabolism. DNA damage and repair mechanisms are also activated, involving DNA helicase activity and nucleotide binding, while cell cycle and DNA replication initiation may be utilized by the host for cellular repair or exploited by the pathogens to promote their replication. These integrated responses constitute the host’s defense strategy against co-infection, aimed at clearing pathogens and repairing damage, but they may also pose risks of tissue damage and chronic inflammation.

Although qRT-PCR is frequently employed as a validation strategy for RNA-seq findings, a recent study indicates that RNA-seq methodologies and analytical pipelines were sufficiently robust and the routine qPCR validation was not obligation—though additional confirmation may still be beneficial in specific contexts (59). Accordingly, this study relied exclusively on high-quality RNA-seq data obtained through rigorous experimental design, adequate sequencing depth, and stringent quality control measures, which can independently serve as a reliable scientific basis for drawing conclusions.

## 5 Conclusion

*E. coli* and *S. aureus* trigger distinct host immune responses—*E. coli* predominantly activates *TLR4*-*NF-κB*-driven inflammation, while *S. aureus* disrupts metabolic and epigenetic regulation. When *E. coli* and *S. aureus* co-infect, the host cell response becomes more complex, involving multiple aspects such as inflammatory responses, metabolic reprogramming, DNA damage and repair, and cell cycle regulation. These findings reveal pathogen-specific defense mechanisms and potential therapeutic targets for bacterial mastitis.

## Data Availability

The raw RNA-sequencing data generated in this study have been deposited in the NCBI Sequence Read Archive (SRA) under BioProject accession PRJNA1264488 (https://www.ncbi.nlm.nih.gov/bioproject/PRJNA1264488). All other data supporting the conclusions of the article are included within the article and its supplementary materials.

## Footnotes

^1^ https://www.bic.ac.cn/BIC/#/

^2^ https://www.ensembl.org/

^3^ https://cn.string-db.org/

## References

[1] Rollin E Dhuyvetter KC Overton MW. The cost of clinical mastitis in the first 30 days of lactation: an economic modeling tool. Prev Vet Med. (2015) 122:257–64. doi: 10.1016/j.prevetmed.2015.11.006, PMID: 26596651

[2] Costa A Bovenhuis H Egger-Danner C Fuerst-Waltl B Boutinaud M Guinard-Flament J. Mastitis has a cumulative and lasting effect on milk yield and lactose content in dairy cows. J Dairy Sci. (2025) 108:635–50. doi: 10.3168/jds.2024-25467, PMID: 39343227

[3] Gonçalves J Freu G Garcia B Barcelos M Alves B Leite R. Effect of bovine subclinical mastitis on milk production and economic performance of Brazilian dairy farms. Braz J Vet Res Anim Sci. (2023) 60:e208514. doi: 10.11606/issn.1678-4456.bjvras.2023.208514

[4] Pascu C Herman V Iancu I Costinar L. Etiology of mastitis and antimicrobial resistance in dairy cattle farms in the western part of Romania. Antibiotics (Basel). (2022) 11:11. doi: 10.3390/antibiotics11010057, PMID: 35052934 PMC8772981

[5] Iancu I Igna V Popa SA Imre K Pascu C Costinar L. Etiology and antimicrobial resistance of subclinical mastitis pathogens *Staphylococcus aureus*, *Streptococcus spp*. and *Enterococcus* spp. in sheep milk. Vet Res Commun. (2024) 49:30. doi: 10.1007/s11259-024-10579-7, PMID: 39576396

[6] Günther J Petzl W Bauer I Ponsuksili S Zerbe H Schuberth H-J. Differentiating *Staphylococcus aureus* from *Escherichia coli* mastitis: *S. aureus* triggers unbalanced immune-dampening and host cell invasion immediately after udder infection. Sci Rep. (2017) 7:4811. doi: 10.1038/s41598-017-05107-4, PMID: 28684793 PMC5500526

[7] Ying Y-T Yang J Tan X Liu R Zhuang Y Xu J-X. *Escherichia coli* and *Staphylococcus aureus* differentially regulate *Nrf2* pathway in bovine mammary epithelial cells: relation to distinct innate immune response. Cells. (2021) 10:3426. doi: 10.3390/cells10123426, PMID: 34943933 PMC8700232

[8] Islam MA Takagi M Fukuyama K Komatsu R Albarracin L Nochi T. Transcriptome analysis of the inflammatory responses of bovine mammary epithelial cells: exploring immunomodulatory target genes for bovine mastitis. Pathogens. (2020) 9:9. doi: 10.3390/pathogens9030200, PMID: 32182886 PMC7157600

[9] Gilbert FB Cunha P Jensen K Glass EJ Foucras G Robert-Granié C. Differential response of bovine mammary epithelial cells to *Staphylococcus aureus* or *Escherichia coli* agonists of the innate immune system. Vet Res. (2013) 44:40. doi: 10.1186/1297-9716-44-40, PMID: 23758654 PMC3686618

[10] Zhuang C Gao J Liu G Zhou M Yang J Wang D. Selenomethionine activates selenoprotein S, suppresses Fas/FasL and the mitochondrial pathway, and reduces *Escherichia coli*-induced apoptosis of bovine mammary epithelial cells. J Dairy Sci. (2021) 104:10171–82. doi: 10.3168/jds.2020-20034, PMID: 34053755

[11] Zhuang C Liu G Barkema HW Zhou M Xu S Ur Rahman S. Selenomethionine suppressed *TLR4*/ *NF-κB* pathway by activating selenoprotein S to alleviate ESBL *Escherichia coli*-induced inflammation in bovine mammary epithelial cells and macrophages. Front Microbiol. (2020) 11:1461. doi: 10.3389/fmicb.2020.01461, PMID: 32733409 PMC7360804

[12] Tao L Liu K Li J Zhang Y Cui L Dong J. Selenomethionine alleviates *NF-κB* -mediated inflammation in bovine mammary epithelial cells induced by *Escherichia coli* by enhancing autophagy. Int Immunopharmacol. (2022) 110:108989. doi: 10.1016/j.intimp.2022.108989, PMID: 35785729

[13] Ma N Chang G Huang J Wang Y Gao Q Cheng X. Cis-9, trans-11-conjugated linoleic acid exerts an anti-inflammatory effect in bovine mammary epithelial cells after *Escherichia coli* stimulation through *NF-κB* signaling pathway. J Agric Food Chem. (2019) 67:193–200. doi: 10.1021/acs.jafc.8b05500, PMID: 30562023

[14] Liu K Zhou X Fang L Dong J Cui L Li J. *PINK1*/parkin-mediated mitophagy alleviates *Staphylococcus aureus*-induced *NLRP3* inflammasome and *NF-κB* pathway activation in bovine mammary epithelial cells. Int Immunopharmacol. (2022) 112:109200. doi: 10.1016/j.intimp.2022.109200, PMID: 36063687

[15] Yang Y Lv S Wang Z Liu J. Selenium ameliorates *S. aureus*-induced inflammation in bovine mammary epithelial cells by regulating ROS-induced *NLRP3* inflammasome. Biol Trace Elem Res. (2022) 200:3171–5. doi: 10.1007/s12011-021-02924-7, PMID: 34535880

[16] Wang X Liu M Geng N Du Y Li Z Gao X. *Staphylococcus aureus* mediates pyroptosis in bovine mammary epithelial cell via activation of *NLRP3* inflammasome. Vet Res. (2022) 53:10. doi: 10.1186/s13567-022-01027-y, PMID: 35123552 PMC8817610

[17] Bolger AM Lohse M Usadel B. Trimmomatic: a flexible trimmer for Illumina sequence data. Bioinformatics. (2014) 30:2114–20. doi: 10.1093/bioinformatics/btu170, PMID: 24695404 PMC4103590

[18] Kim D Paggi JM Park C Bennett C Salzberg SL. Graph-based genome alignment and genotyping with HISAT2 and HISAT-genotype. Nat Biotechnol. (2019) 37:907–15. doi: 10.1038/s41587-019-0201-4, PMID: 31375807 PMC7605509

[19] Li H Handsaker B Wysoker A Fennell T Ruan J Homer N. The sequence alignment/map format and Samtools. Bioinformatics. (2009) 25:2078–9. doi: 10.1093/bioinformatics/btp352, PMID: 19505943 PMC2723002

[20] Pertea M Pertea GM Antonescu CM Chang TC Mendell JT Salzberg SL. StringTie enables improved reconstruction of a transcriptome from RNA-seq reads. Nat Biotechnol. (2015) 33:290–5. doi: 10.1038/nbt.3122, PMID: 25690850 PMC4643835

[21] Chen T Liu Y-X Chen T Yang M Fan S Shi M. Imagegp 2 for enhanced data visualization and reproducible analysis in biomedical research. iMeta. (2024) 3:e239. doi: 10.1002/imt2.239, PMID: 39429882 PMC11487545

[22] Harrison PW Amode MR Austine-Orimoloye O Azov AG Barba M Barnes I. Ensembl 2024. Nucleic Acids Res. (2024) 52:D891–9. doi: 10.1093/nar/gkad1049, PMID: 37953337 PMC10767893

[23] Love MI Huber W Anders S. Moderated estimation of fold change and dispersion for RNA-seq data with DESeq2. Genome Biol. (2014) 15:550. doi: 10.1186/s13059-014-0550-8, PMID: 25516281 PMC4302049

[24] Szklarczyk D Nastou K Koutrouli M Kirsch R Mehryary F Hachilif R. The STRING database in 2025: protein networks with directionality of regulation. Nucleic Acids Res. (2025) 53:D730–7. doi: 10.1093/nar/gkae1113, PMID: 39558183 PMC11701646

[25] Shannon P Markiel A Ozier O Baliga NS Wang JT Ramage D. Cytoscape: a software environment for integrated models of biomolecular interaction networks. Genome Res. (2003) 13:2498–504. doi: 10.1101/gr.1239303, PMID: 14597658 PMC403769

[26] Li C Yin L He X Jin Y Zhu X Wu R. Competition-cooperation mechanism between *Escherichia coli* and *Staphylococcus aureus* based on systems mapping. Front Microbiol. (2023) 14:1192574. doi: 10.3389/fmicb.2023.1192574, PMID: 38029174 PMC10657823

[27] Di Marco F Nicola F Giannese F Saliu F Tonon G de Pretis S. Dual spatial host-bacterial gene expression in *Mycobacterium abscessus* respiratory infections. Commun Biol. (2024) 7:1287. doi: 10.1038/s42003-024-06929-5, PMID: 39384974 PMC11479615

[28] Doxey AC Abu Mazen N Homm M Chu V Hunjan M Lobb B. Metatranscriptomic profiling reveals pathogen and host response signatures of pediatric acute sinusitis and upper respiratory infection. Genome Med. (2025) 17:22. doi: 10.1186/s13073-025-01447-3, PMID: 40098147 PMC11912616

[29] Yang Q Guo H Li H Li Z Ni F Wen Z. The *CXCL8*/*MAPK*/*hnRNP-K* axis enables susceptibility to infection by EV-D68, rhinovirus, and influenza virus *in vitro*. Nat Commun. (2025) 16:1715. doi: 10.1038/s41467-025-57094-0, PMID: 39962077 PMC11832783

[30] Sharifi S Pakdel A Ebrahimi M Reecy JM Fazeli Farsani S Ebrahimie E. Integration of machine learning and meta-analysis identifies the transcriptomic bio-signature of mastitis disease in cattle. PLoS One. (2018) 13:e0191227. doi: 10.1371/journal.pone.0191227, PMID: 29470489 PMC5823400

[31] Han H. Identification of several key genes by microarray data analysis of bovine mammary gland epithelial cells challenged with *Escherichia coli* and *Staphylococcus aureus*. Gene. (2019) 683:123–32. doi: 10.1016/j.gene.2018.10.004, PMID: 30291872

[32] Guo Q Jin Y Chen X Ye X Shen X Lin M. *NF-κB* in biology and targeted therapy: new insights and translational implications. Signal Transduct Target Ther. (2024) 9:53. doi: 10.1038/s41392-024-01757-9, PMID: 38433280 PMC10910037

[33] Li Q Laumonnier Y Syrovets T Simmet T. Recruitment of *CCR6*-expressing Th17 cells by *CCL20* secreted from plasmin-stimulated macrophages. Acta Biochim Biophys Sin Shanghai. (2013) 45:593–600. doi: 10.1093/abbs/gmt049, PMID: 23681234

[34] Wang J Yang J Xia W Zhang M Tang H Wang K. *Escherichia coli* enhances Th17/Treg imbalance via *TLR4*/*NF-κB* signaling pathway in oral lichen planus. Int Immunopharmacol. (2023) 119:110175. doi: 10.1016/j.intimp.2023.110175, PMID: 37058754

[35] Luo R Yao Y Chen Z Sun X. An examination of the LPS-*TLR4* immune response through the analysis of molecular structures and protein-protein interactions. Cell Commun Signal. (2025) 23:142. doi: 10.1186/s12964-025-02149-4, PMID: 40102851 PMC11921546

[36] Rocha SF Schiller M Jing D Li H Butz S Vestweber D. *Esm1* modulates endothelial tip cell behavior and vascular permeability by enhancing VEGF bioavailability. Circ Res. (2014) 115:581–90. doi: 10.1161/circresaha.115.304718, PMID: 25057127

[37] Kelley N Jeltema D Duan Y He Y. The *NLRP3* inflammasome: an overview of mechanisms of activation and regulation. Int J Mol Sci. (2019) 20:20. doi: 10.3390/ijms20133328, PMID: 31284572 PMC6651423

[38] Schmidt RL Lenz LL. Distinct licensing of *IL-18* and *IL-1β* secretion in response to *NLRP3* inflammasome activation. PLoS One. (2012) 7:e45186. doi: 10.1371/journal.pone.0045186, PMID: 23028835 PMC3445464

[39] Kowalski A Pałyga J. Modulation of chromatin function through linker histone H1 variants. Biol Cell. (2016) 108:339–56. doi: 10.1111/boc.201600007, PMID: 27412812

[40] Schultheiß C Willscher E Paschold L Gottschick C Klee B Henkes SS. The *IL-1β*, *IL-6*, and *TNF* cytokine triad is associated with post-acute sequelae of COVID-19. Cell Rep Med. (2022) 3:100663. doi: 10.1016/j.xcrm.2022.100663, PMID: 35732153 PMC9214726

[41] Ranasinghe R Eri R. Modulation of the *CCR6*-*CCL20* axis: a potential therapeutic target in inflammation and cancer. Medicina (Kaunas). (2018) 54:88. doi: 10.3390/medicina54050088, PMID: 30453514 PMC6262638

[42] Lee AY Eri R Lyons AB Grimm MC Korner H. CC chemokine ligand 20 and its cognate receptor *CCR6* in mucosal T cell immunology and inflammatory bowel disease: odd couple or axis of evil? Front Immunol. (2013) 4:194. doi: 10.3389/fimmu.2013.00194, PMID: 23874340 PMC3711275

[43] Zonis S Breunig JJ Mamelak A Wawrowsky K Bresee C Ginzburg N. Inflammation-induced *Gro1* triggers senescence in neuronal progenitors: effects of estradiol. J Neuroinflammation. (2018) 15:260. doi: 10.1186/s12974-018-1298-y, PMID: 30201019 PMC6131894

[44] Michalski C Cheung C Oh JH Ackermann E Popescu CR Archambault AS. *DDIT4L* regulates mitochondrial and innate immune activities in early life. JCI Insight. (2024) 9:e172312. doi: 10.1172/jci.insight.172312, PMID: 38319716 PMC11143921

[45] Chen CA Strouz K Huang KL Shyu AB. *Tob2* phosphorylation regulates global mRNA turnover to reshape transcriptome and impact cell proliferation. RNA. (2020) 26:1143–59. doi: 10.1261/rna.073528.119, PMID: 32404348 PMC7430666

[46] Jozefczuk S Klie S Catchpole G Szymanski J Cuadros-Inostroza A Steinhauser D. Metabolomic and transcriptomic stress response of *Escherichia coli*. Mol Syst Biol. (2010) 6:364. doi: 10.1038/msb.2010.18, PMID: 20461071 PMC2890322

[47] Leto TL Morand S Hurt D Ueyama T. Targeting and regulation of reactive oxygen species generation by Nox family NADPH oxidases. Antioxid Redox Signal. (2009) 11:2607–19. doi: 10.1089/ars.2009.2637, PMID: 19438290 PMC2782575

[48] Latini FR Hemerly JP Oler G Riggins GJ Cerutti JM. Re-expression of *ABI3*-binding protein suppresses thyroid tumor growth by promoting senescence and inhibiting invasion. Endocr Relat Cancer. (2008) 15:787–99. doi: 10.1677/erc-08-0079, PMID: 18559958 PMC2742300

[49] Feng Y Tao F Qiao H Tang H. A pan-cancer analysis of *ABI3BP*: a potential biomarker for prognosis and immunoinfiltration. Front Oncol. (2023) 13:1159725. doi: 10.3389/fonc.2023.1159725, PMID: 37197424 PMC10183607

[50] Feng Y Han X Zhang Z Qiao H Tang H. *ABI3BP* is a prognosis biomarker related with clinicopathological features and immunity infiltration of lung tumor. Front Genet. (2022) 13:1085785. doi: 10.3389/fgene.2022.1085785 36744181 PMC9894588

[51] E HS Bradshaw AD Brekken R. SPARC, a matricellular protein that regulates cell-matrix interaction: implications for vascular and connective tissue biology In: Oakazaki I Ninomiya Y Friedman SL Tanikawa K, editors. Extracellular matrix and the liver. San Diego: Academic Press (2003). 75–85.

[52] Brzozowski JS Skelding KA. The multi-functional calcium/calmodulin stimulated protein kinase (CaMk) family: emerging targets fo anti-cancer therapeutic intervention. Pharmaceuticals (Basel). (2019) 12:8. doi: 10.3390/ph12010008, PMID: 30621060 PMC6469190

[53] Kondo Y Ledderose C Slubowski CJ Fakhari M Sumi Y Sueyoshi K. Frontline science: *Escherichia coli* use LPS as decoy to impair neutrophil chemotaxis and defeat antimicrobial host defense. J Leukoc Biol. (2019) 106:1211–9. doi: 10.1002/jlb.4hi0319-109r, PMID: 31392789 PMC6883117

[54] Long N Deng J Qiu M Zhang Y Wang Y Guo W. Inflammatory and pathological changes in *Escherichia coli* infected mice. Heliyon. (2022) 8:e12533. doi: 10.1016/j.heliyon.2022.e12533, PMID: 36643320 PMC9834738

[55] Pakbin B Brück WM Rossen JWA. Virulence factors of enteric pathogenic *Escherichia coli*: a review. Int J Mol Sci. (2021) 22:22. doi: 10.3390/ijms22189922, PMID: 34576083 PMC8468683

[56] Zhao J Yang W Gao B Wang H Chen L Shan C. *Escherichia coli* HPI-induced duodenitis through ubiquitin regulation of the *TLR4*/*NF-κB* pathway. BMC Vet Res. (2025) 21:66. doi: 10.1186/s12917-025-04515-3, PMID: 39953596 PMC11829554

[57] Thakore PI Schnell A Huang L Zhao M Hou Y Christian E. *BACH2* regulates diversification of regulatory and proinflammatory chromatin states in TH17 cells. Nat Immunol. (2024) 25:1395–410. doi: 10.1038/s41590-024-01901-1, PMID: 39009838

[58] Ha KP Edwards AM. DNA repair in *Staphylococcus aureus*. Microbiol Mol Biol Rev. (2021) 85:e0009121. doi: 10.1128/mmbr.00091-21, PMID: 34523959 PMC8483670

[59] Coenye T. Do results obtained with RNA-sequencing require independent verification? Biofilms. (2021) 3:100043. doi: 10.1016/j.bioflm.2021.100043, PMID: 33665610 PMC7823214

