# Supplementary material for: Mechanisms of host-bacterial interactions during Escherichia coli or Staphylococcus aureus infection of mammary epithelial cells
Source: Front Vet Sci. 2025 Jul 23;12:1644768. doi: 10.3389/fvets.2025.1644768 (PMC12327436; doi:10.3389/fvets.2025.1644768)
Supplement: Supplementary file 1 [file Table_1.DOCX]

Supplementary Material

# Supplementary Figures and Tables

## Supplementary Figures


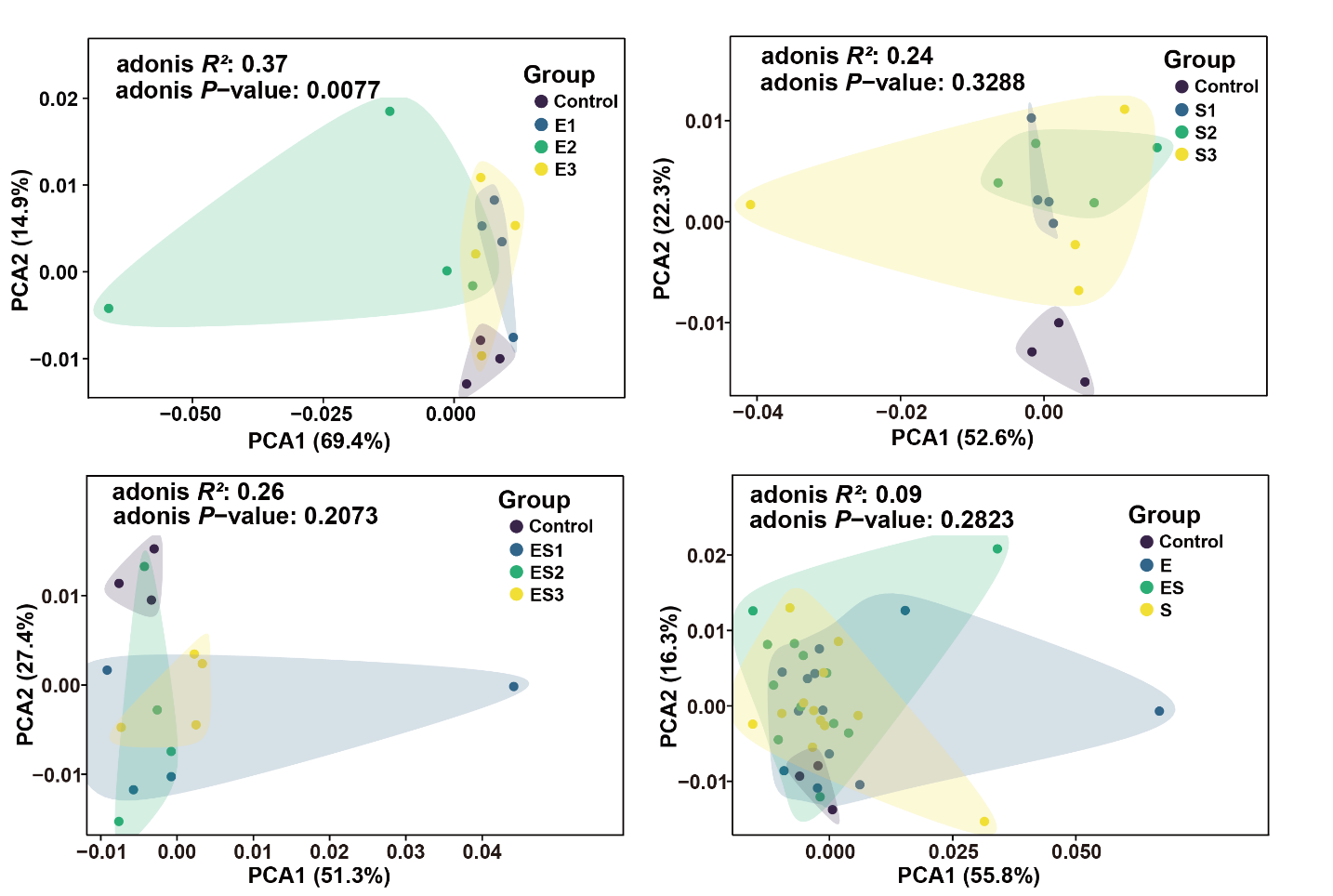


**Supplementary Figure 1.** Principal component analysis (PCA) of different infection groups
E: *Escherichia coli* infection group. E1, E2, and E3 are different infection concentration groups, with the concentration decreasing from front to back.

ES: Co-infection group of *Escherichia coli* and *Staphylococcus aureus*. ES1, ES2, and ES3 are different infection concentration groups, with the concentration decreasing from front to back.

S: *Staphylococcus aureus* infection group. S1, S2, and S3 are different infection concentration groups, with the concentration decreasing from front to back.

**
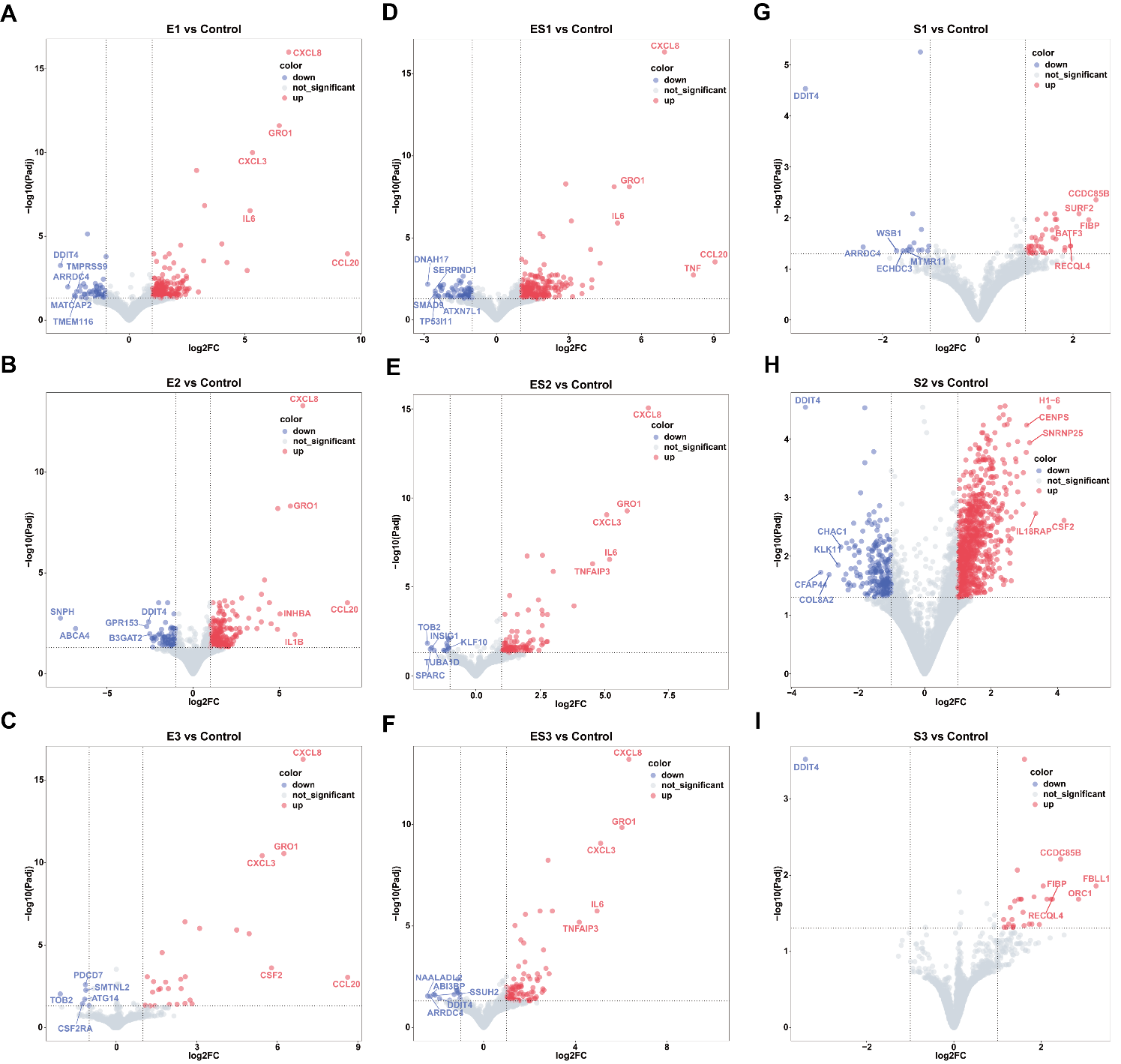
**

**Supplementary Figure 2.** Volcano plots of treatment groups vs. Control
The volcano plot illustrates the distribution of differentially expressed genes (DEGs) in each treatment group compared to the control, with the top 5 upregulated and downregulated DEGs specifically labeled.


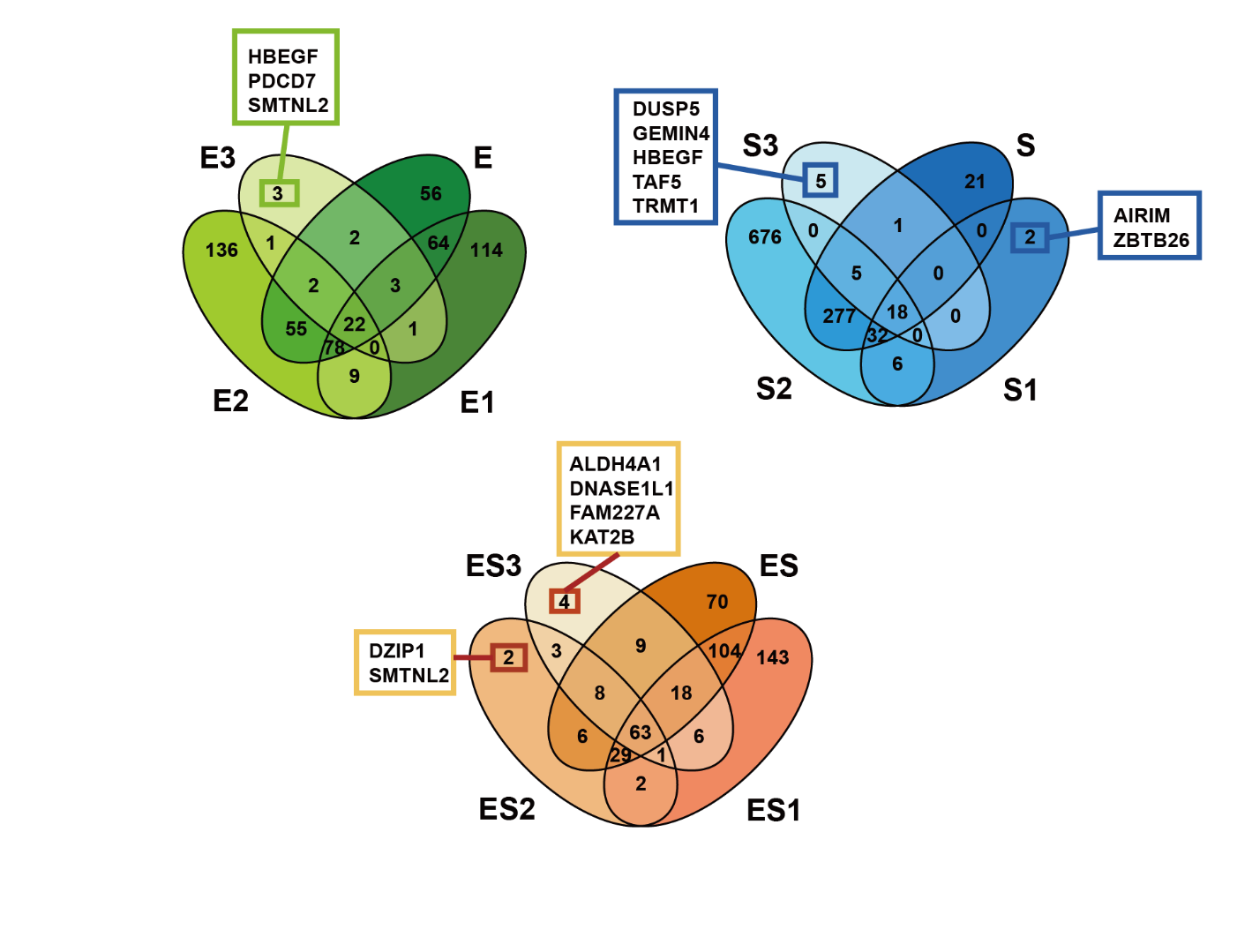


**Supplementary Figure 3.** The Venn diagram of different infection groups


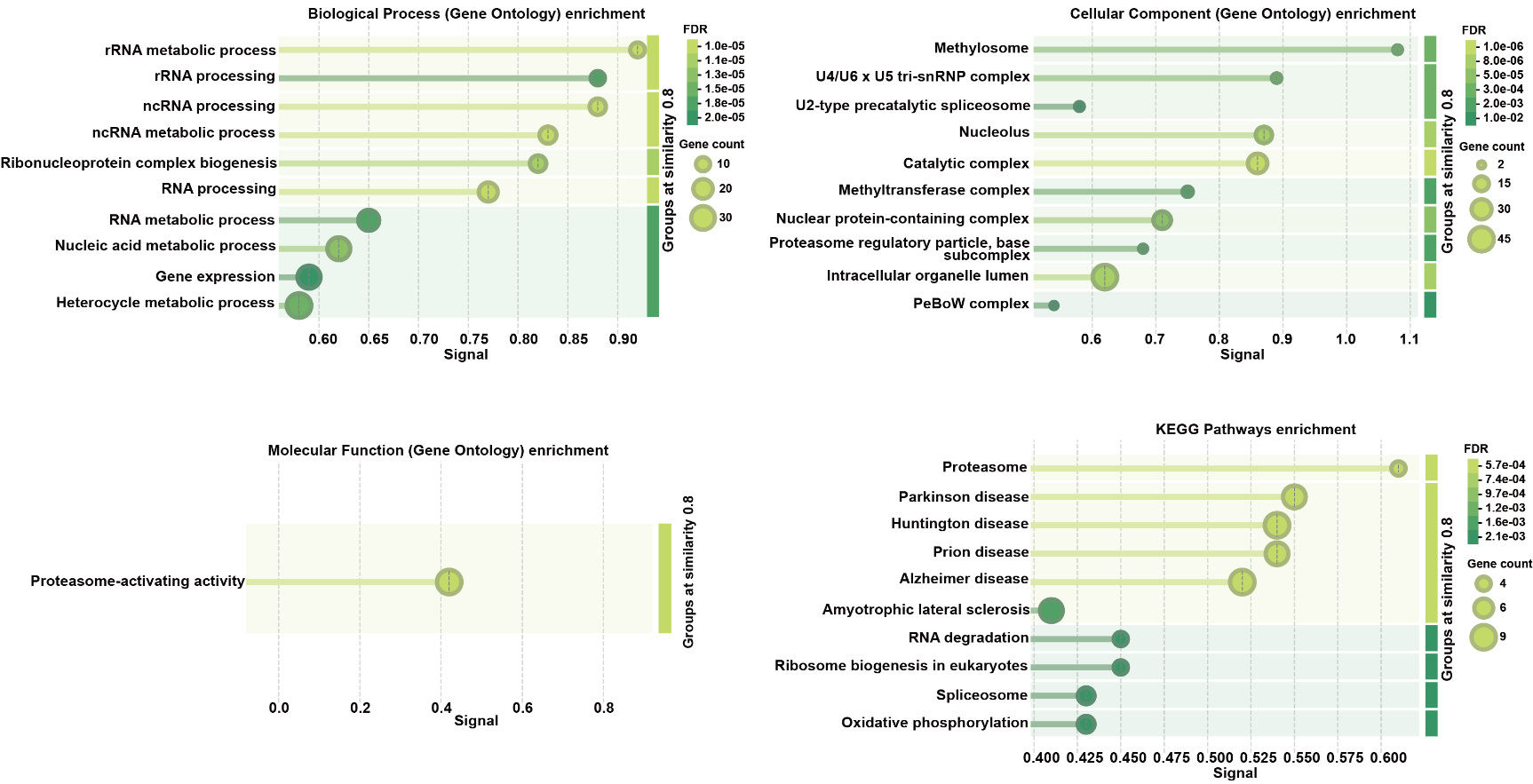


**Supplementary Figure 4.** Functional enrichment results of E1-specific genes in the *Escherichia coli* infection group


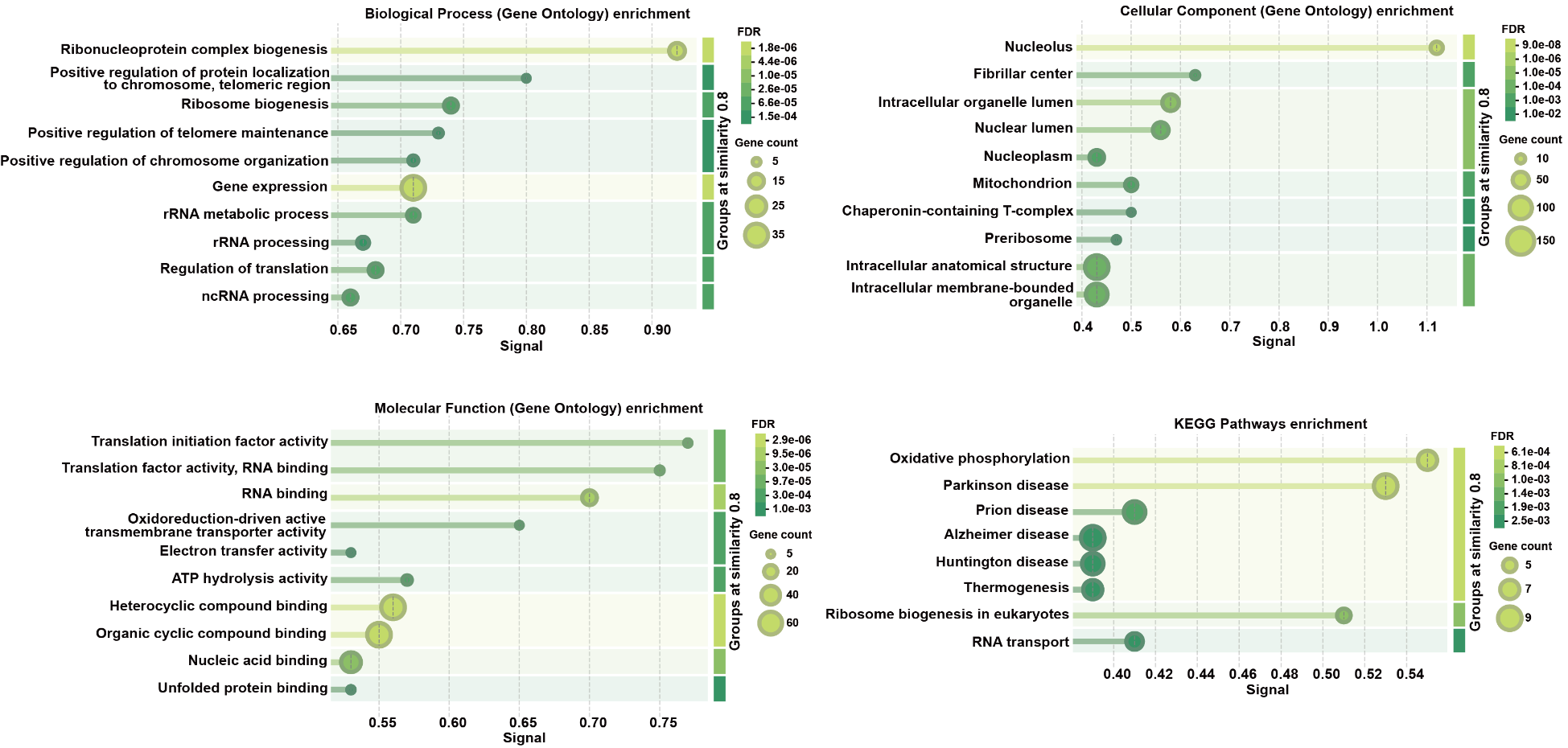


**Supplementary Figure 5.** Functional enrichment results of E2-specific genes in the *Escherichia coli* infection group


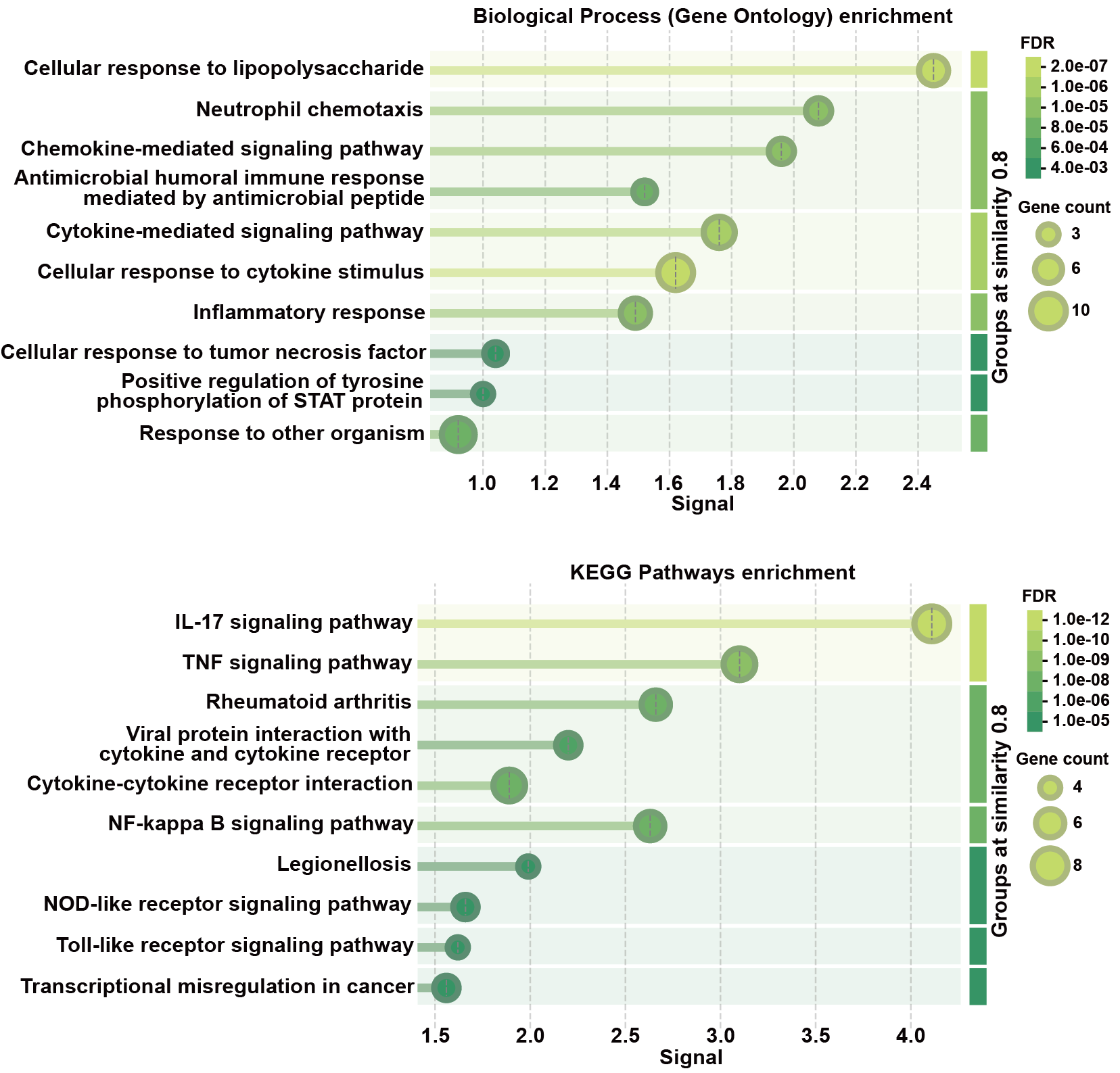


**Supplementary Figure 6.** Functional enrichment results of E1-, E2-, E3-, and E-shared genes in the *Escherichia coli* infection group


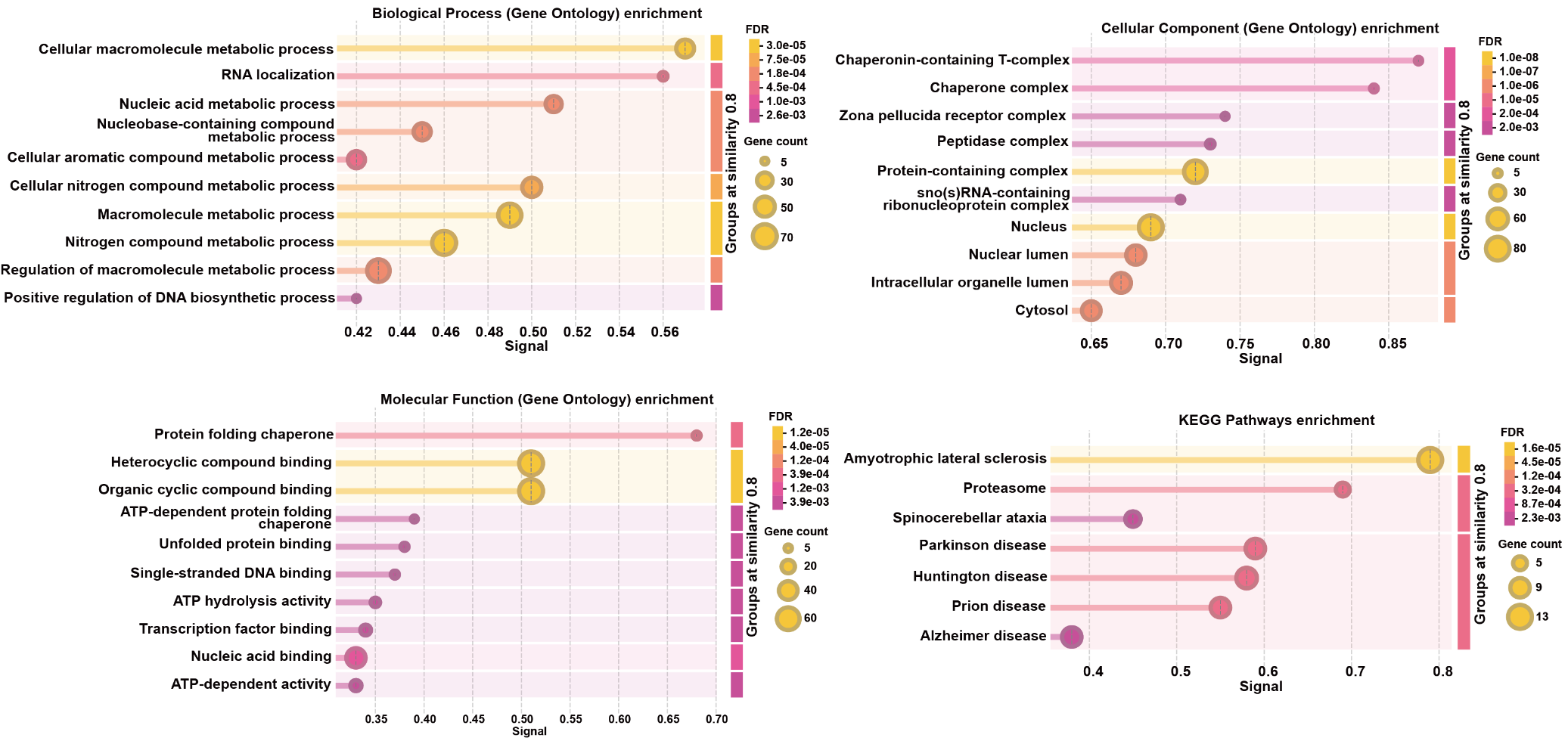


**Supplementary Figure 7.** Functional enrichment results of ES1-specific genes in the co-infection group of *Escherichia coli* and *Staphylococcus aureus*


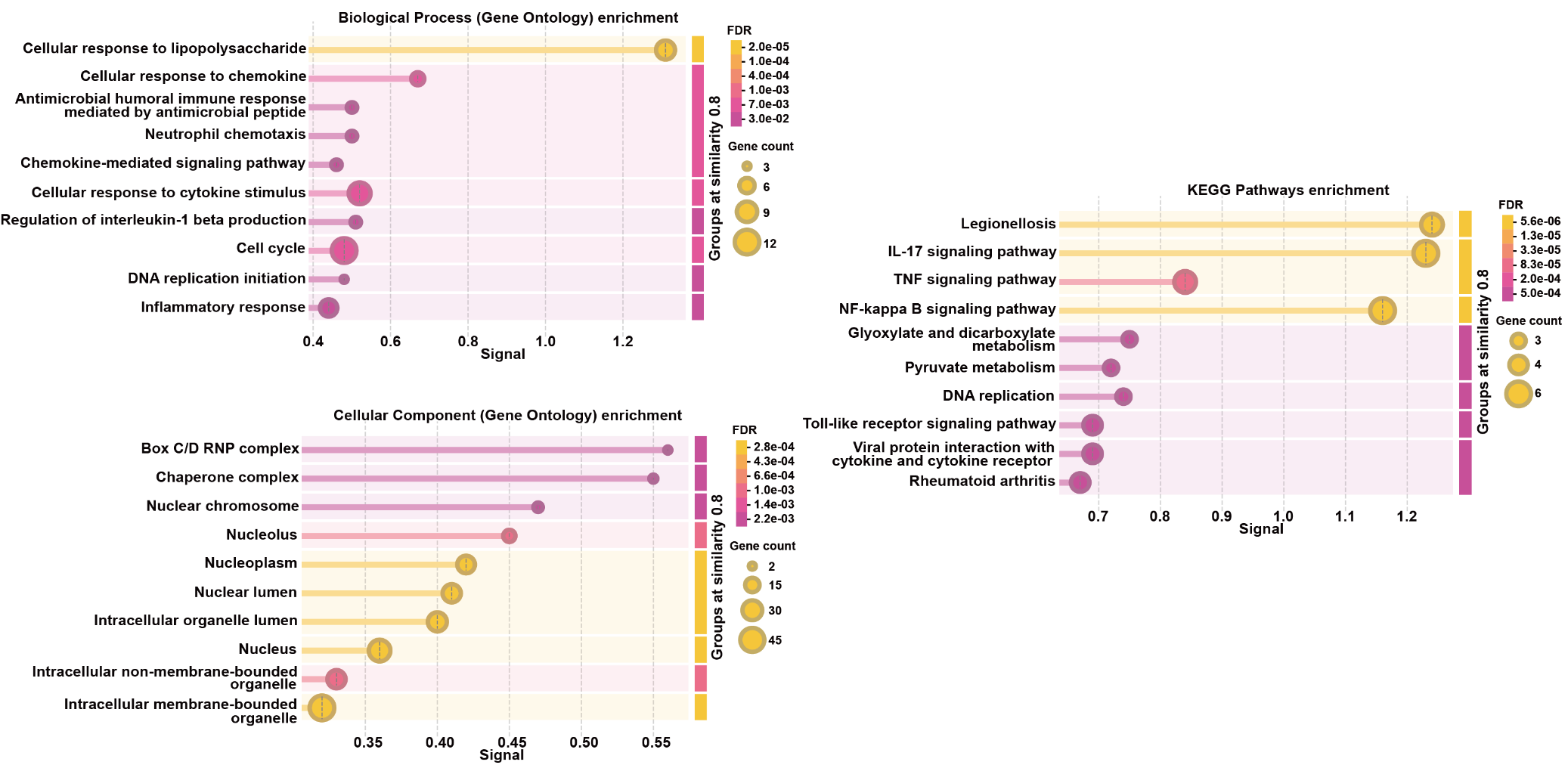


**Supplementary Figure 8.** Functional enrichment results of ES1-, ES2-, ES3-, and ES-shared genes in the co-infection group of *Escherichia coli* and *Staphylococcus aureus*


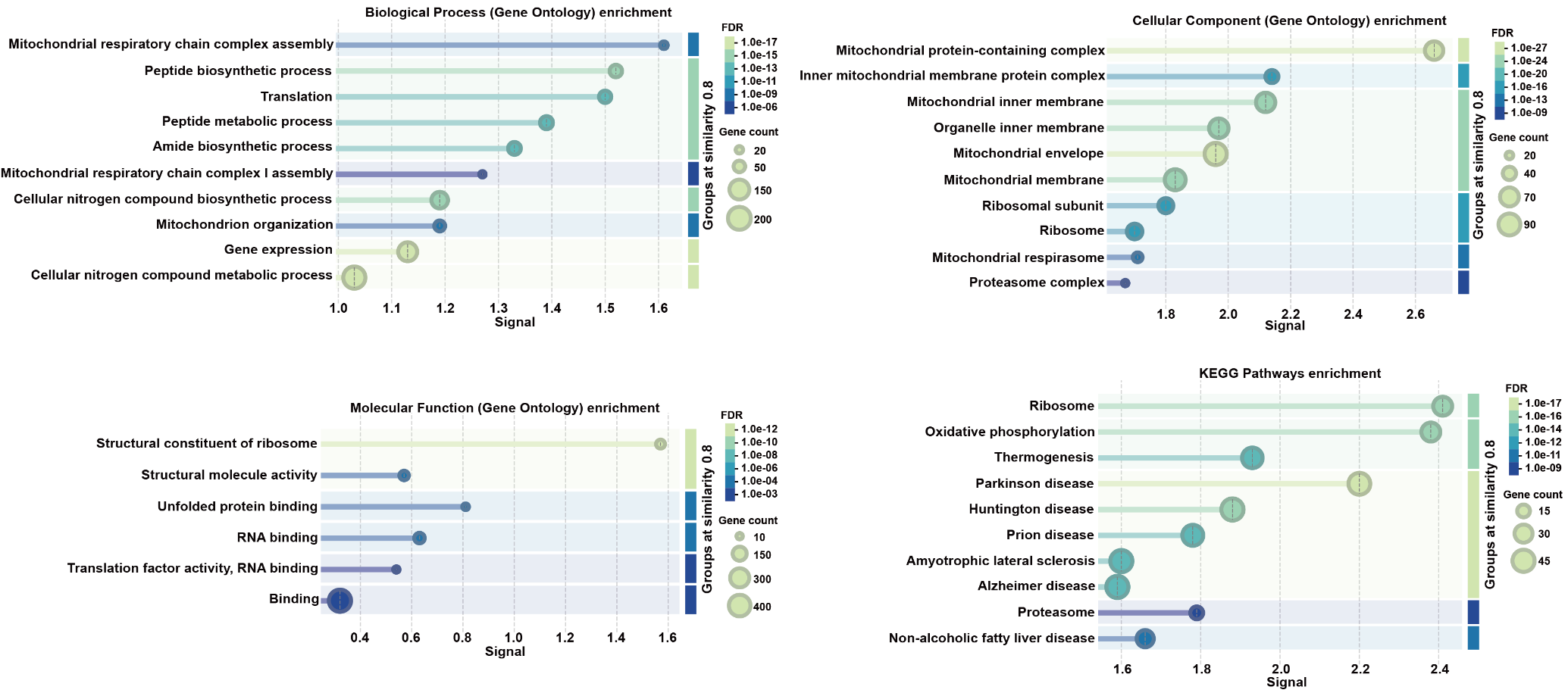


**Supplementary Figure 9.** Functional enrichment results of S2-specific genes in the *Staphylococcus aureus* infection group


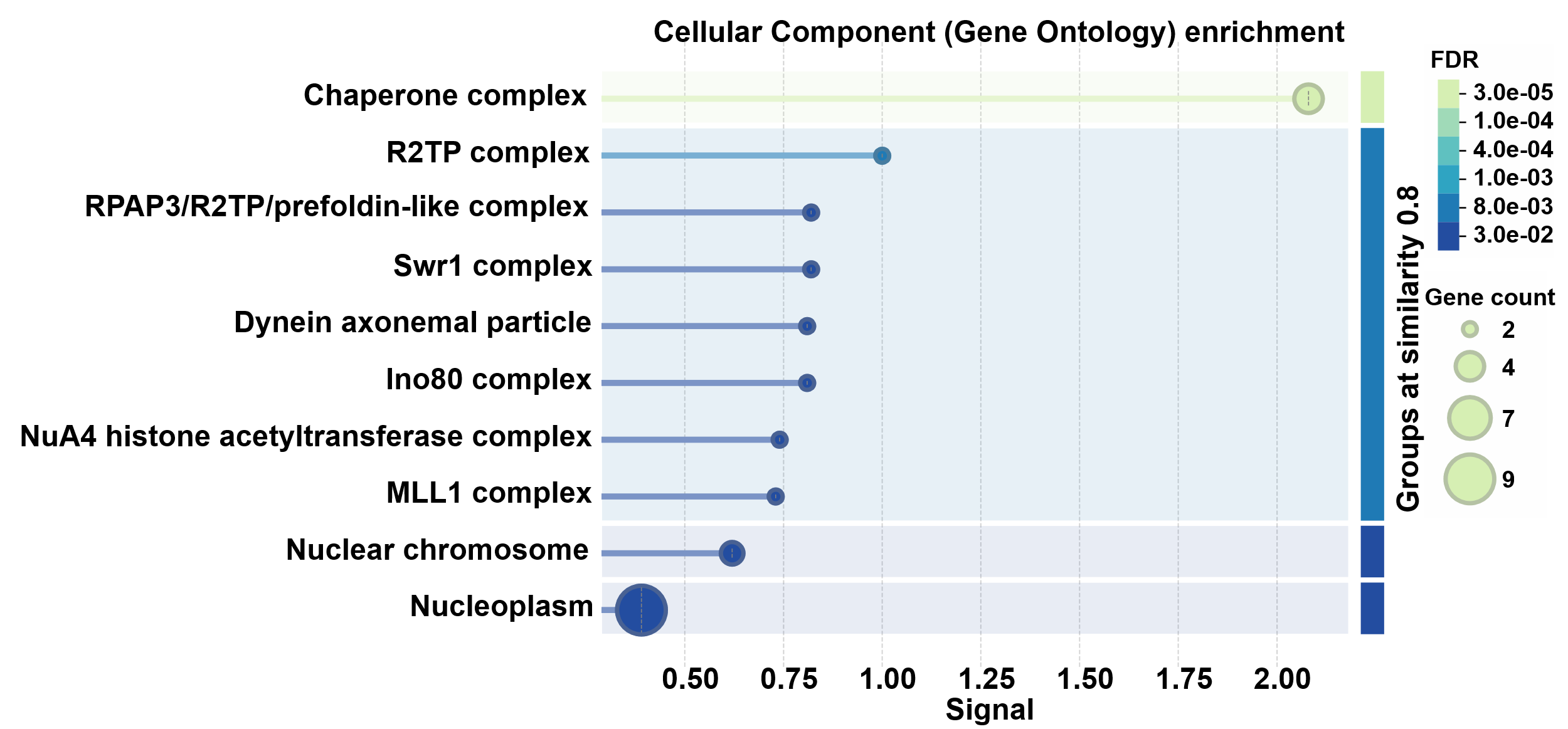


**Supplementary Figure 10.** Functional enrichment results of S1-, S2-, S3-, and S-shared genes in the *Staphylococcus aureus* infection group


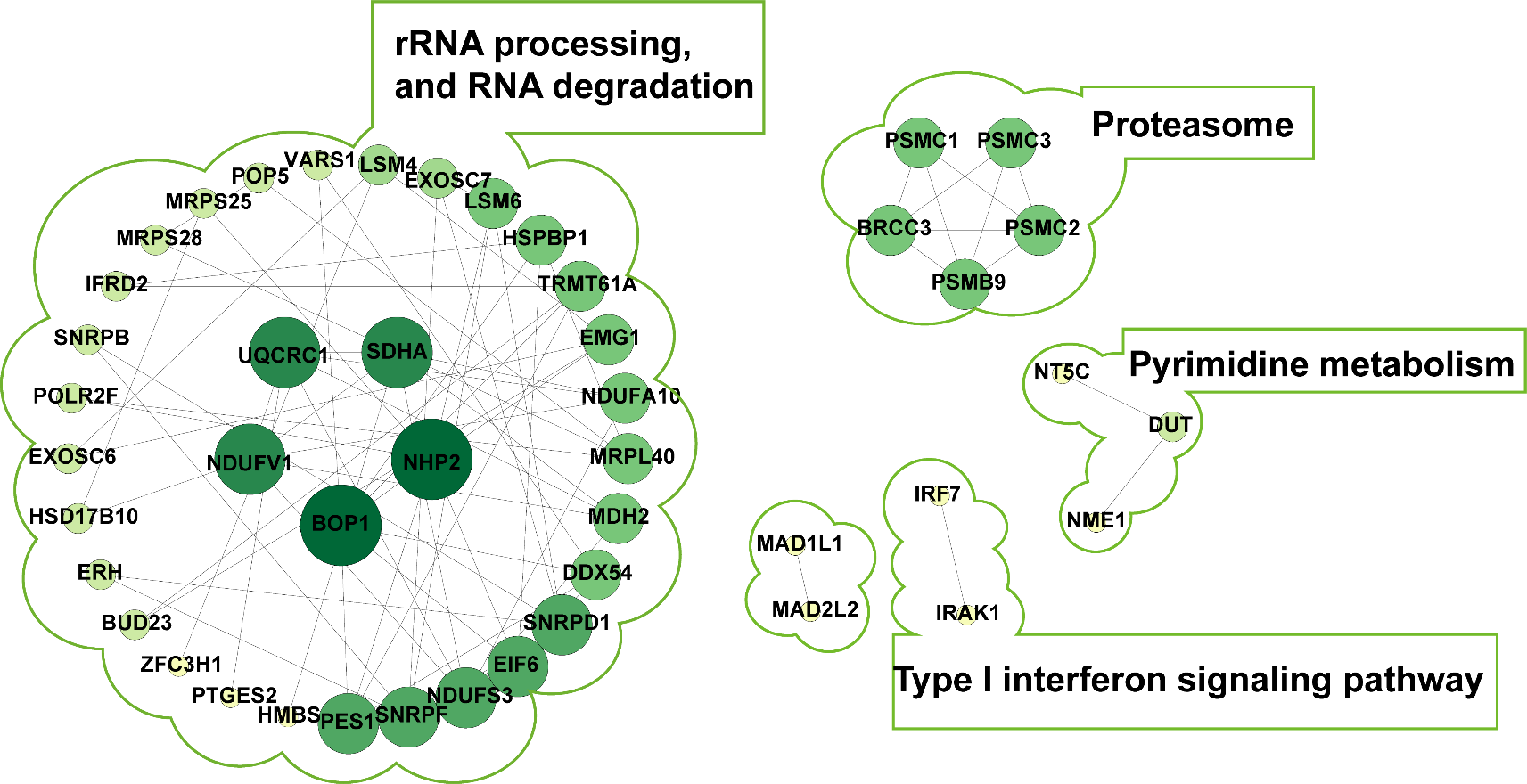


**Supplementary Figure 11.** Protein-protein interaction (PPI) network of uniquely expressed genes in the E1 treatment group


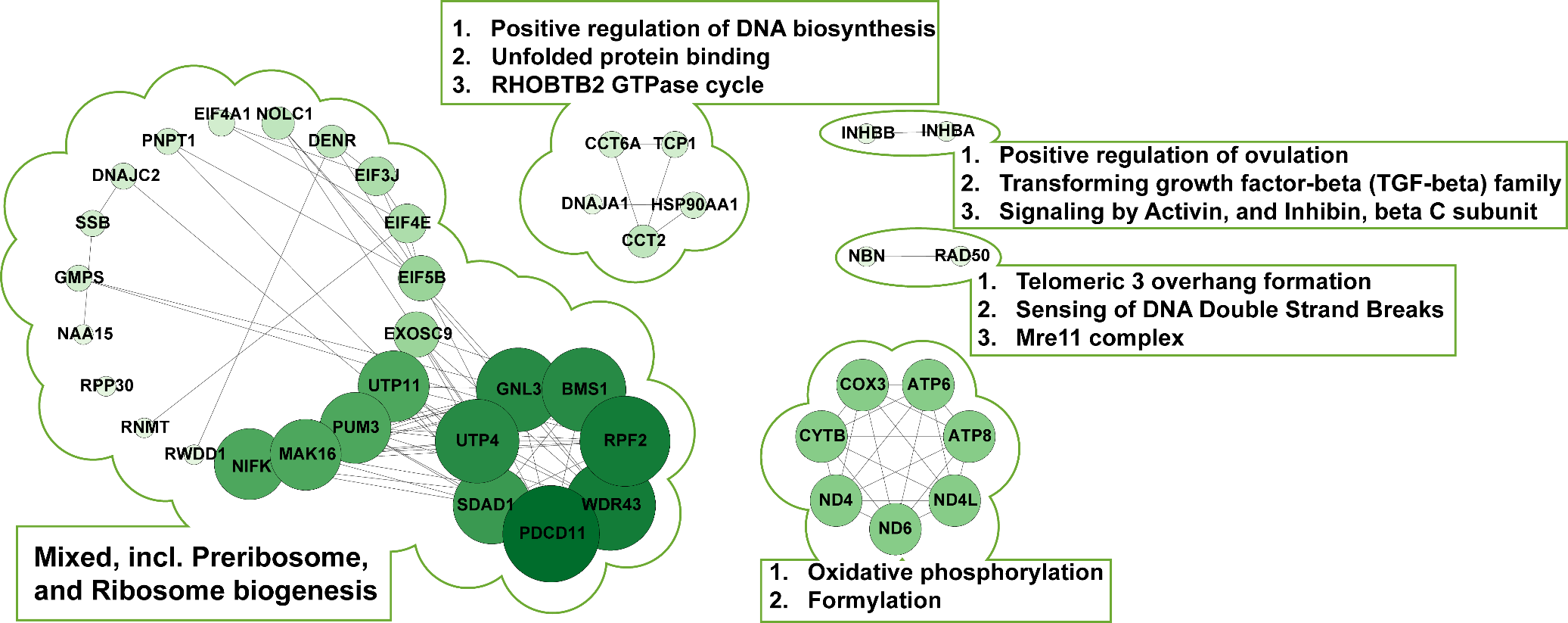


**Supplementary Figure 12.** Protein-protein interaction (PPI) network of uniquely expressed genes in the E2 treatment group


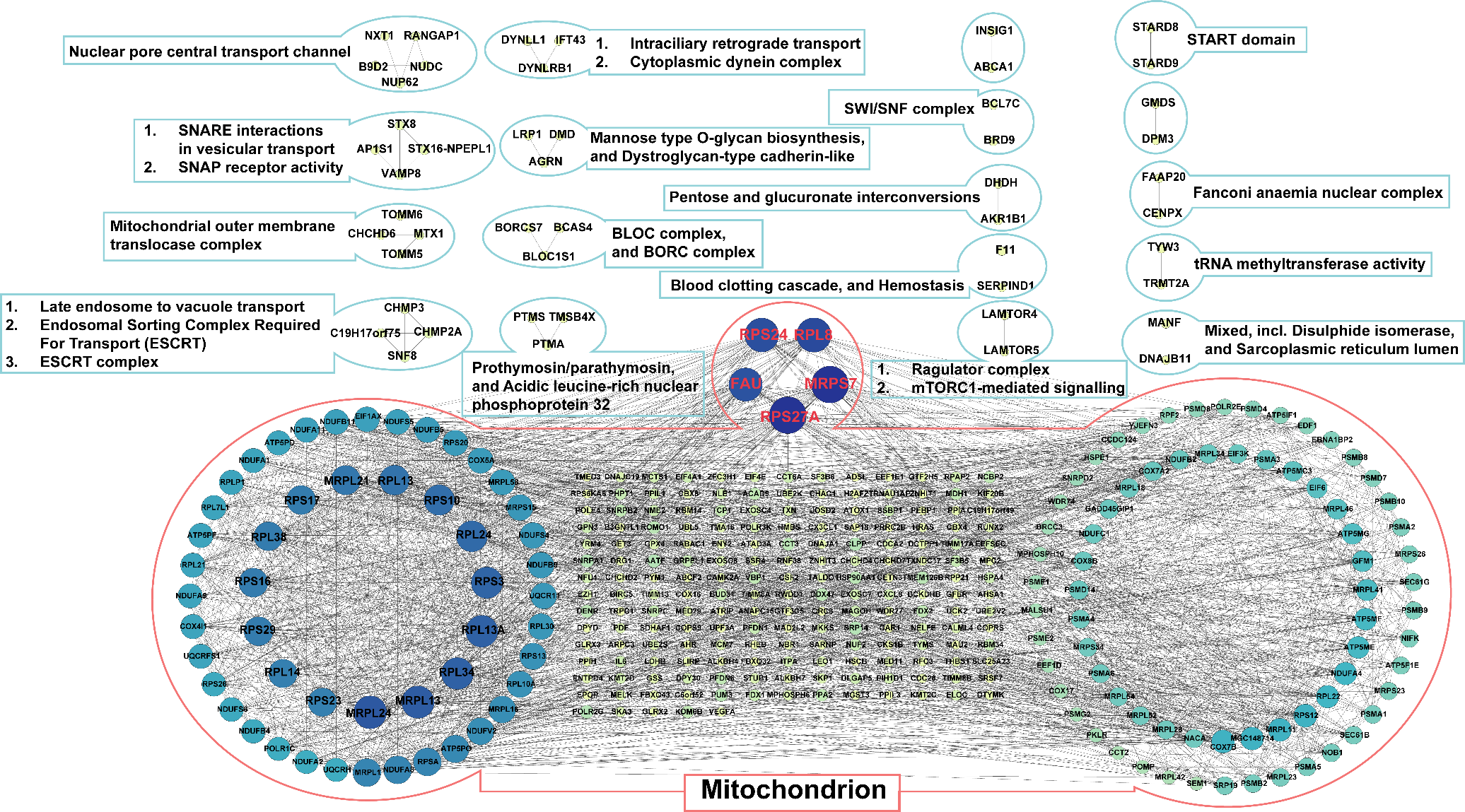


**Supplementary Figure 13.** Protein-protein interaction (PPI) network of uniquely expressed genes in the S2 treatment group


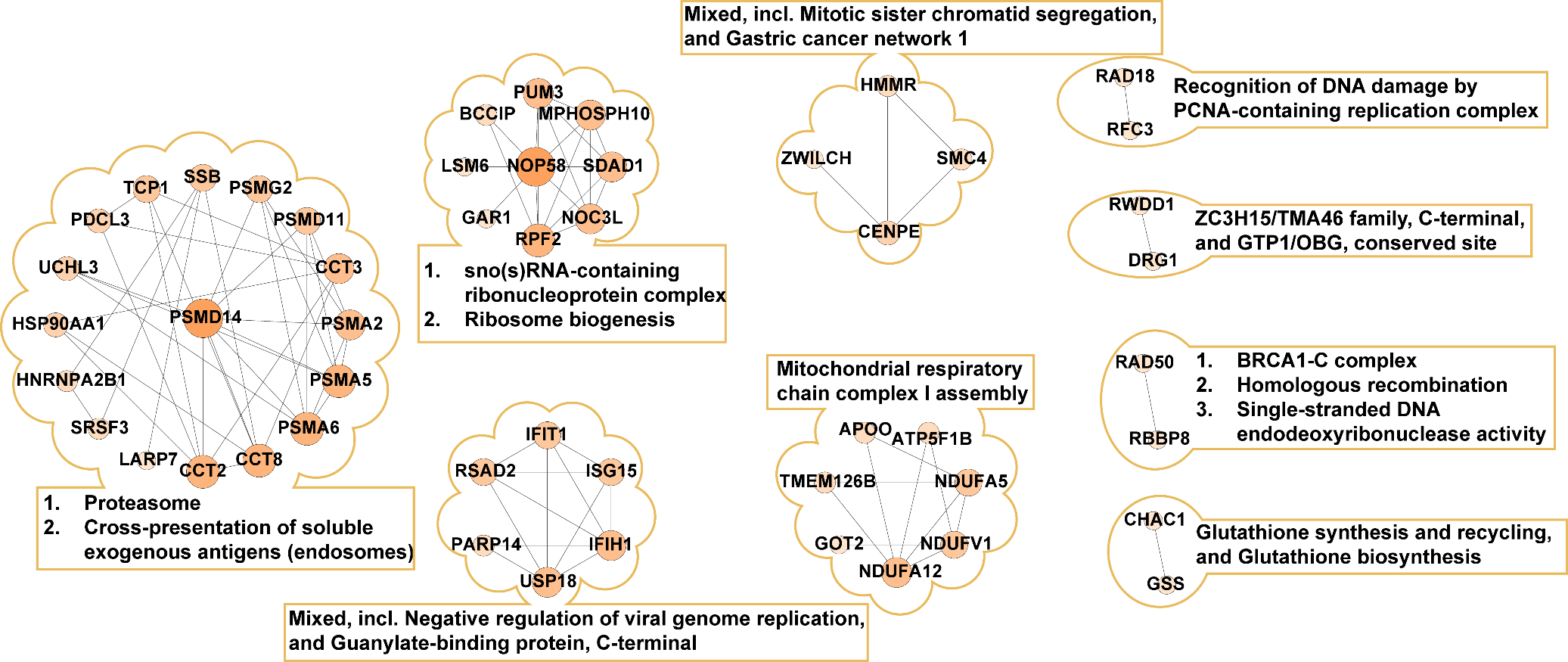


**Supplementary Figure 14.** Protein-protein interaction (PPI) network of uniquely expressed genes in the ES1 treatment group

## Supplementary Tables

| Trimmomatic | | | HISAT2 | |
| --- | --- | --- | --- | --- |
| Sample | Input Read Pairs | Both Surviving | Input | Overall Alignment Rate |
| DZ1 | 20240261 | 19315162 (95.43%) | 19315162 | 95.69% |
| DZ2 | 19364055 | 18523180 (95.66%) | 18523180 | 95.30% |
| DZ3 | 18957317 | 18194301 (95.98%) | 18194301 | 95.33% |
| E1-2T | 18978729 | 18238695 (96.10%) | 18238695 | 95.64% |
| E1-6T | 19538471 | 18697876 (95.70%) | 18697876 | 96.68% |
| E1-10T | 19741156 | 18887856 (95.68%) | 18887856 | 96.41% |
| E1-24T | 20941945 | 20039830 (95.69%) | 20039830 | 96.12% |
| E2-2T | 20765943 | 19905598 (95.86%) | 19905598 | 97.16% |
| E2-6T | 21158030 | 20312486 (96.00%) | 20312486 | 96.56% |
| E2-10T | 19150892 | 18306716 (95.59%) | 18306716 | 96.62% |
| E2-24T | 20604032 | 19638146 (95.31%) | 19638146 | 97.06% |
| E3-2T | 18853516 | 18097462 (95.99%) | 18097462 | 95.32% |
| E3-6T | 19170669 | 18321338 (95.57%) | 18321338 | 95.21% |
| E3-10T | 20519744 | 19622611 (95.63%) | 19622611 | 95.08% |
| E3-24T | 21146223 | 20209605 (95.57%) | 20209605 | 95.39% |
| ES1-2T | 18904788 | 18075593 (95.61%) | 18075593 | 95.49% |
| ES1-6T | 20451272 | 19625801 (95.96%) | 19625801 | 96.74% |
| ES1-10T | 20486475 | 19583339 (95.59%) | 19583339 | 94.79% |
| ES1-24T | 20696112 | 19628880 (94.84%) | 19628880 | 95.86% |
| ES2-2T | 20275481 | 19423943 (95.80%) | 19423943 | 95.77% |
| ES2-6T | 19245697 | 18303071 (95.10%) | 18303071 | 95.07% |
| ES2-10T | 19925915 | 19104753 (95.88%) | 19104753 | 96.82% |
| ES2-24T | 20895011 | 19887596 (95.18%) | 19887596 | 96.66% |
| ES3-2T | 19445310 | 18492149 (95.10%) | 18492149 | 96.48% |
| ES3-6T | 18650076 | 17934261 (96.16%) | 17934261 | 94.74% |
| ES3-10T | 19918386 | 18990249 (95.34%) | 18990249 | 95.49% |
| ES3-24T | 21122596 | 20146333 (95.38%) | 20146333 | 94.72% |
| S1-2T | 19205683 | 18406770 (95.84%) | 18406770 | 95.82% |
| S1-6T | 20221598 | 19337295 (95.63%) | 19337295 | 96.31% |
| S1-10T | 20454734 | 19533841 (95.50%) | 19533841 | 96.96% |
| S1-24T | 23437794 | 22509919 (96.04%) | 22509919 | 96.69% |
| S2-2T | 21772987 | 20734031 (95.23%) | 20734031 | 97.43% |
| S2-6T | 19413784 | 18515202 (95.37%) | 18515202 | 96.49% |
| S2-10T | 19205157 | 18374797 (95.68%) | 18374797 | 95.54% |
| S2-24T | 24508982 | 23473768 (95.78%) | 23473768 | 96.21% |
| S3-2T | 21111724 | 20171004 (95.54%) | 20171004 | 95.30% |
| S3-6T | 18476249 | 17416834 (94.27%) | 17416834 | 96.36% |
| S3-10T | 21352898 | 20336578 (95.24%) | 20336578 | 94.65% |
| S3-24T | 23621480 | 22514026 (95.31%) | 22514026 | 94.72% |
| Sum | 791931172 | 756830895 |  |  |

**Supplementary Table 1.** The results of quality control and alignment for 39 samples

Quality Score Format: Phred33

The parameter SLIDINGWINDOW:4:20 indicates a window size of 4 bases. If the average quality score within any position of the sliding window falls below 20, all bases at the end of the window will be trimmed.

The parameter MINLEN:60 specifies that the trimmed sequences must contain at least 60 bases. Sequences that do not meet this length requirement will be discarded.

The genome assembly ARS-UCD1.3 was downloaded from the Ensembl website for Bos taurus. The specific file used was Bos_taurus.ARS-UCD1.3.dna_sm.toplevel.fa. The quality-controlled sequences were then aligned to this reference genome. The overall alignment rate, which indicates the proportion of sequences that successfully aligned to the reference genome, was a key metric in assessing the alignment quality.

| Pairs | Df | SumsOfSqs | F.Model | R2 | P.value | P.adjusted |
| --- | --- | --- | --- | --- | --- | --- |
| Control vs E | 1 | 0.0007 | 1.2627 | 0.0885 | 0.2045 | 0.4090 |
| Control vs ES | 1 | 0.0007 | 2.3784 | 0.1547 | 0.1245 | 0.3735 |
| Control vs S | 1 | 0.0007 | 2.4142 | 0.1566 | 0.0903 | 0.3735 |
| E vs ES | 1 | 0.0005 | 0.9747 | 0.0424 | 0.4233 | 0.5922 |
| E vs S | 1 | 0.0003 | 0.5996 | 0.0265 | 0.6955 | 0.6955 |
| ES vs S | 1 | 0.0003 | 0.8530 | 0.0373 | 0.4935 | 0.5922 |
| Control vs E1 | 1 | 0.0005 | 4.6666 | 0.4828 | 0.0286 | 0.0571 |
| Control vs E2 | 1 | 0.0015 | 1.9055 | 0.2759 | 0.1429 | 0.1714 |
| Control vs E3 | 1 | 0.0004 | 2.9579 | 0.3717 | 0.0857 | 0.1286 |
| E1 vs E2 | 1 | 0.0016 | 2.3994 | 0.2857 | 0.0258 | 0.0571 |
| E1 vs E3 | 1 | 0.0001 | 0.7953 | 0.1170 | 0.5910 | 0.5910 |
| E2 vs E3 | 1 | 0.0014 | 2.0680 | 0.2563 | 0.0278 | 0.0571 |
| Control vs S1 | 1 | 0.0005 | 6.2581 | 0.5559 | 0.0286 | 0.0857 |
| Control vs S2 | 1 | 0.0006 | 3.5214 | 0.4132 | 0.0286 | 0.0857 |
| Control vs S3 | 1 | 0.0004 | 0.9737 | 0.1630 | 0.5429 | 0.8782 |
| S1 vs S2 | 1 | 0.0001 | 0.7732 | 0.1142 | 0.6575 | 0.8782 |
| S1 vs S3 | 1 | 0.0001 | 0.2389 | 0.0383 | 1.0000 | 1.0000 |
| S2 vs S3 | 1 | 0.0003 | 0.6191 | 0.0935 | 0.7318 | 0.8782 |
| Control vs ES1 | 1 | 0.0008 | 1.7309 | 0.2572 | 0.0571 | 0.1714 |
| Control vs ES2 | 1 | 0.0005 | 2.6438 | 0.3459 | 0.1143 | 0.2286 |
| Control vs ES3 | 1 | 0.0004 | 4.2414 | 0.4590 | 0.0286 | 0.1714 |
| ES1 vs ES2 | 1 | 0.0003 | 0.6067 | 0.0918 | 0.8892 | 0.8892 |
| ES1 vs ES3 | 1 | 0.0003 | 0.6428 | 0.0968 | 0.7830 | 0.8892 |
| ES2 vs ES3 | 1 | 0.0001 | 0.7779 | 0.1148 | 0.6018 | 0.8892 |

**Supplementary Table 2.** PERMANOVA (adonis) test for differences

| Group | E | E1 | E2 | E3 | S | S1 | S2 | S3 | ES | ES1 | ES2 | ES3 |
| --- | --- | --- | --- | --- | --- | --- | --- | --- | --- | --- | --- | --- |
| Sig_genes | 282 | 291 | 303 | 34 | 354 | 58 | 1014 | 29 | 307 | 366 | 114 | 112 |
| Up | 246 | 234 | 231 | 28 | 314 | 42 | 784 | 28 | 277 | 294 | 101 | 99 |
| Down | 36 | 57 | 72 | 6 | 40 | 16 | 220 | 1 | 30 | 72 | 13 | 13 |

**Supplementary Table 3.** Differentially expressed genes (DEGs) counts by treatment group

E: *Escherichia coli* infection group. E1, E2, and E3 are different infection concentration groups, with the concentration decreasing from front to back.

ES: Co-infection group of *Escherichia coli* and *Staphylococcus aureus*. ES1, ES2, and ES3 are different infection concentration groups, with the concentration decreasing from front to back.

S: *Staphylococcus aureus* infection group. S1, S2, and S3 are different infection concentration groups, with the concentration decreasing from front to back.

| Treatment | Down | Up |
| --- | --- | --- |
| E | ABI3BP, ECHDC3, NYNRIN, TOB2, DDIT4 | CXCL8, GRO1, CCL20, TNF, IL-1B |
| E1 | DDIT4, ARRDC4, MATCAP2, TMEM116, TMPRSS9 | IL6, CXCL3, GRO1, CXCL8, CCL20 |
| E2 | SNPH, ABCA4, GPR153, DDIT4, B3GAT2 | INHBA, GRO1, IL1B, CXCL8, CCL20 |
| E3 | TOB2, CSF2RA, ATG14, PDCD7, SMTNL2 | CXCL3, CSF2, GRO1, CXCL8, CCL20 |
| ES | SPARC, CAMK2A, TOB2, PCLO, SSUH2 | CCL20, TNF, CXCL8, GRO1, IL6 |
| ES1 | DNAH17, SERPIND1, TP53I11, SMAD9, ATXN7L1 | IL6, GRO1, CXCL8, TNF, CCL20 |
| ES2 | TOB2, SPARC, INSIG1, TUBA1D, KLF10 | TNFAIP3, CXCL3, IL6, GRO1, CXCL8 |
| ES3 | NAALADL2, ARRDC4, ABI3BP, SSUH2, DDIT4 | TNFAIP3, IL6, CXCL3, GRO1, CXCL8 |
| S | ABI3BP, NOXO1, SSUH2, PCLO, DDIT4 | ESM1, IL18RAP, H1-6, GRO1, ORC1 |
| S1 | DDIT4, ARRDC4, WSB1, ECHDC3, MTMR11 | RECQL4, BATF3, SURF2, FIBP, CCDC85B |
| S2 | DDIT4, CFAP44, COL8A2, KLK11, CHAC1 | CENPS, SNRNP25, IL18RAP, H1-6, CSF2 |
| S3 | DDIT4 | FIBP, RECQL4, CCDC85B, ORC1, FBLL1 |

**Supplementary Table 4.** Top 5 up- and down-regulated differentially expressed genes (DEGs) in each treatment group

E: *Escherichia coli* infection group. E1, E2, and E3 are different infection concentration groups, with the concentration decreasing from front to back. ES: Co-infection group of *Escherichia coli* and *Staphylococcus aureus*. ES1, ES2, and ES3 are different infection concentration groups, with the concentration decreasing from front to back. S: *Staphylococcus aureus* infection group. S1, S2, and S3 are different infection concentration groups, with the concentration decreasing from front to back.

| Treatment | number of nodes | number of edges | average node degree | avg. local clustering coefficient | expected number of edges | PPI enrichment *P*-value |
| --- | --- | --- | --- | --- | --- | --- |
| E1 | 107 | 74 | 1.38 | 0.286 | 29 | 1.56E-12 |
| E2 | 133 | 110 | 1.65 | 0.327 | 37 | ＜1.0E-16 |
| E1∩E2∩E3∩E | 21 | 25 | 2.38 | 0.383 | 1 | ＜1.0E-16 |
| ES1 | 140 | 85 | 1.21 | 0.311 | 30 | 1.11E-16 |
| ES1∩ES2∩ES3∩E | 60 | 43 | 1.43 | 0.29 | 12 | 1.70E-12 |
| S2 | 647 | 2183 | 6.75 | 0.389 | 1367 | ＜1.0E-16 |
| S1∩S2∩S3∩S | 16 | 2 | 0.25 | 0.125 | 0 | 0.0157 |

**Supplementary Table 5.** Network Stats information on the String website

E1, E2, ES1, S2 input are their unique genes, while E1∩E2∩E3∩E, ES1∩ES2∩ES3∩E, S1∩S2∩S3∩S input are their shared genes.
